# Different Characteristics and Nucleotide Binding Properties of Inosine Monophosphate Dehydrogenase (IMPDH) Isoforms

**DOI:** 10.1371/journal.pone.0051096

**Published:** 2012-12-07

**Authors:** Elaine C. Thomas, Jennifer H. Gunter, Julie A. Webster, Nicole L. Schieber, Viola Oorschot, Robert G. Parton, Jonathan P. Whitehead

**Affiliations:** 1 Diamantina Institute for Cancer, Immunology and Metabolic Medicine, Princess Alexandra Hospital, University of Queensland, Brisbane, Queensland, Australia; 2 Metabolic Medicine, Mater Medical Research Institute, South Brisbane, Queensland, Australia; 3 The University of Queensland, Institute for Molecular Bioscience, Brisbane, Queensland, Australia; University of Technology Sydney, Australia

## Abstract

We recently reported that Inosine Monophosphate Dehydrogenase (IMPDH), a rate-limiting enzyme in *de novo* guanine nucleotide biosynthesis, clustered into macrostructures in response to decreased nucleotide levels and that there were differences between the IMPDH isoforms, IMPDH1 and IMPDH2. We hypothesised that the Bateman domains, which are present in both isoforms and serve as energy-sensing/allosteric modules in unrelated proteins, would contribute to isoform-specific differences and that mutations situated in and around this domain in *IMPDH1* which give rise to retinitis pigmentosa (RP) would compromise regulation. We employed immuno-electron microscopy to investigate the ultrastructure of IMPDH macrostructures and live-cell imaging to follow clustering of an IMPDH2-GFP chimera in real-time. Using a series of IMPDH1/IMPDH2 chimera we demonstrated that the propensity to cluster was conferred by the N-terminal 244 amino acids, which includes the Bateman domain. A protease protection assay suggested isoform-specific purine nucleotide binding characteristics, with ATP protecting IMPDH1 and AMP protecting IMPDH2, via a mechanism involving conformational changes upon nucleotide binding to the Bateman domain without affecting IMPDH catalytic activity. ATP binding to IMPDH1 was confirmed in a nucleotide binding assay. The RP-causing mutation, R224P, abolished ATP binding and nucleotide protection and this correlated with an altered propensity to cluster. Collectively these data demonstrate that (i) the isoforms are differentially regulated by AMP and ATP by a mechanism involving the Bateman domain, (ii) communication occurs between the Bateman and catalytic domains and (iii) the RP-causing mutations compromise such regulation. These findings support the idea that the IMPDH isoforms are subject to distinct regulation and that regulatory defects contribute to human disease.

## Introduction

Inosine monophosphate dehydrogenase (IMPDH) catalyses the rate-limiting step in the *de novo* biosynthesis of guanine nucleotides, which are essential for various cellular processes. In mammals there are two ubiquitously expressed IMPDH isoforms, termed IMPDH1 and IMPDH2, which are encoded by distinct genes [1], [2]. The proteins share 84% amino acid identity and virtually indistinguishable catalytic activity, as determined *in vitro*, but differ in their tissue expression [1], [2], [3], [4]. In most tissues IMPDH2 is the dominant isoform and is up-regulated in proliferating cells and down-regulated upon differentiation [1], [2], [5], [6]. IMPDH1 is typically expressed at low levels but mRNA levels are high in tissues including pancreas, brain, kidney and spleen [6]. Interestingly, mutations in the *IMPDH1* gene, but not *IMPDH2*, give rise to a number of closely related retinal diseases [7], [8]. To begin to investigate this specificity we characterised the spatio-temporal expression of IMPDH isoforms in the developing rat retina [9]. We found that retinal expression of IMPDH was complex, with IMPDH2 being the predominant isoform during the early stages of development and IMPDH1, and variants thereof, being the major species following eye opening [9]. We also observed striking differences in the propensity of IMPDH1 and IMPDH2 to cluster into filamentous spicules (1–2 µm) and ‘macrostructures’ (2–10 µm), however, the two isoforms responded similarly to gross changes in intracellular nucleotide levels [9]. Given the high degree of similarity between IMPDH1 and IMPDH2 proteins we found the former observation particularly intriguing. We hypothesised that divergence in the tandem cystathionine β-synthase (CBS) domain-containing Bateman domain underpinned these differences and also that disease-causing mutations situated in or around the Bateman domain compromised the regulation of IMPDH1 rather than its activity *per se* [10], [11], [12].

Bateman domains are conserved throughout evolution and are present in a wide number of proteins [13], [14] most notably the master cellular energy regulator AMP kinase (AMPK) [15]. Typically, they are not required for catalytic activity but provide important allosteric regulation [16]. The physiological and pathophysiological significance of these domains is highlighted by the finding that mutations within the Bateman domains of a number of proteins, in addition to IMPDH1, are associated with disease [16]. Notwithstanding this, the function of the Bateman domain in IMPDH remains controversial. It is not essential for enzymatic activity or tetramerisation [17], [18] and crystallization studies indicate that the Bateman domains project away from the catalytic core of the IMPDH tetramer [19], [20]. Recent studies by Pimkin and Markham (2008) have demonstrated the *in vivo* importance of the IMPDH Bateman domain in the regulation of nucleotide homeostasis [21]. The authors found *guaB* ^ΔCBS^ *E.coli*, which have the Bateman domain deleted from the chromosomal *guaB* (bacterial IMPDH) gene, were unable to maintain normal ATP and GTP pools, particularly in response to metabolic challenges with exogenous purine bases [21]. Indeed, allosteric regulation of IMPDH by ATP had been proposed by Scott and colleagues (2004); who observed ATP bound to purified human IMPDH2, via the Bateman domain, with positive cooperativity which resulted in a four-fold increase in catalytic activity [22]. However, other studies have been unable to recapitulate this allosteric activation of IMPDH by ATP [3], [12], [21], [23]. Interestingly, purified human IMPDH2 protein has also been reported to bind GTP, at physiological concentrations, although GTP had little effect on catalytic activity [23].

In the current report we aimed to investigate a putative role for the Bateman domain in IMPDH clustering [9], [23] and to establish whether differences in the Bateman domains underpinned the different clustering properties of the IMPDH isoforms. We also aimed to gain a greater understanding of the nucleotide binding properties of both IMPDH isoforms and the effects of the two most prevalent disease-causing mutations, R224P and D226N [7], [8], on the properties of IMPDH1.

## Results

### Characterisation of IMPDH Macrostructures

**Figure 1:**
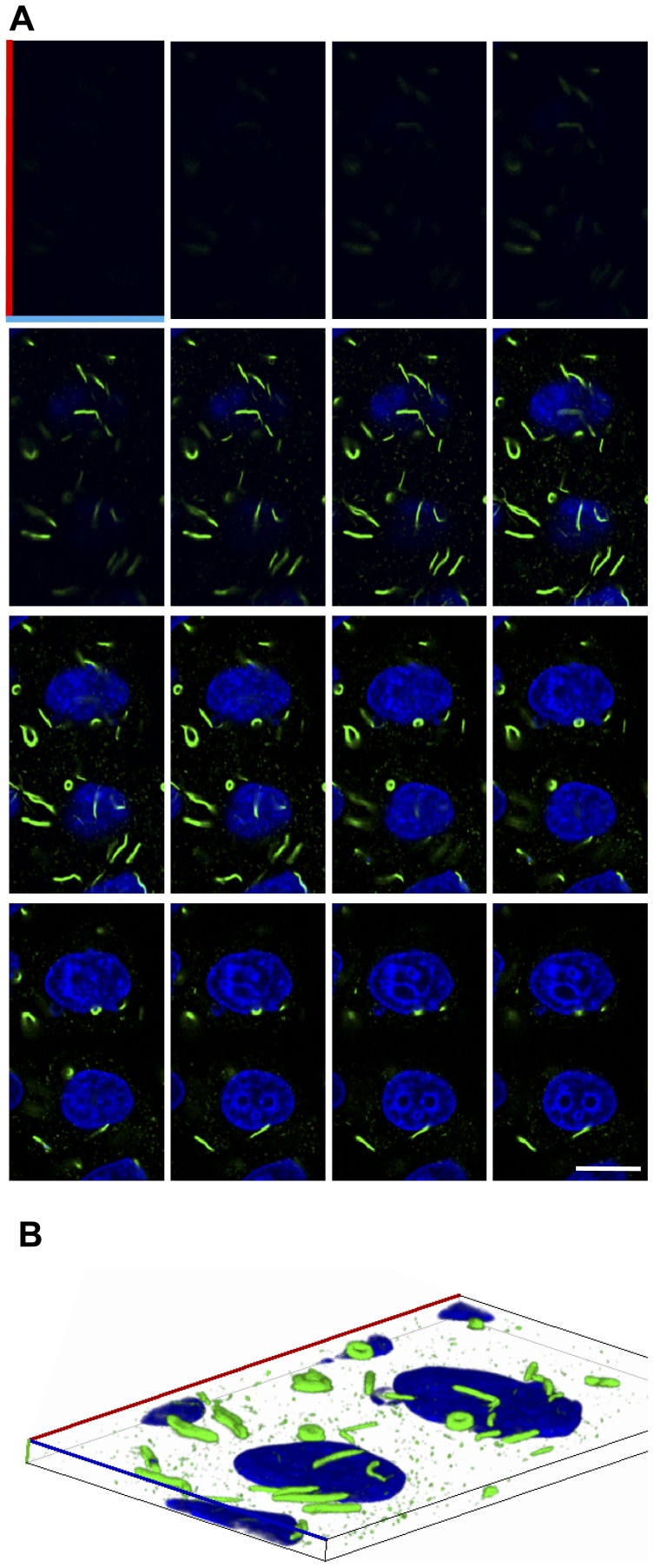
Optical sections of IMPDH macrostructures. Representative confocal z-series of HeLa cells treated with 1 µM MPA for 4 h. Cells were fixed, permeabilised and labelled with the anti-panIMPDH antibody (green) and nuclei were counterstained with DAPI (blue). (A) Montage of images from apical (top left) to basal (bottom right) are spaced by increments of 0.539 µm on the z-axis. Scale bar = 20 µm. (B) Stacked images were viewed with Zeiss Zen 2007 software using the transparency rendering mode in the 3-dimension function. The plane of the cells was rotated to view macrostructures wrapping around the nucleus and the tops of the “donut” macrostructures. Axis' are coloured to orientate to the top left image in A.

We previously demonstrated that redistribution of IMPDH to macrostructures may be promoted upon intracellular nucleotide depletion with mycophenolic acid (MPA), an IMPDH inhibitor, and more importantly with decoyinine, a specific inhibitor of the enzyme GMP synthetase which catalyses the reaction immediately downstream of IMPDH [9]. To further delineate the organisation of IMPDH macrostructures within cells, MPA-treated HeLa cells were optically sectioned by confocal z-series (Fig. 1). Macrostructures were located predominantly in the apical layers of the cell as shown in the montage of slices (Fig. 1A). The macrostructures were consistently in the perinuclear region, wrapping around the nucleus in all dimensions (Fig. 1B). The three-dimensional image also shows that the circular/annular macrostructures are true “donuts” and do not represent solid spheres with a core which is inaccessible to antibody.

**Figure 2:**
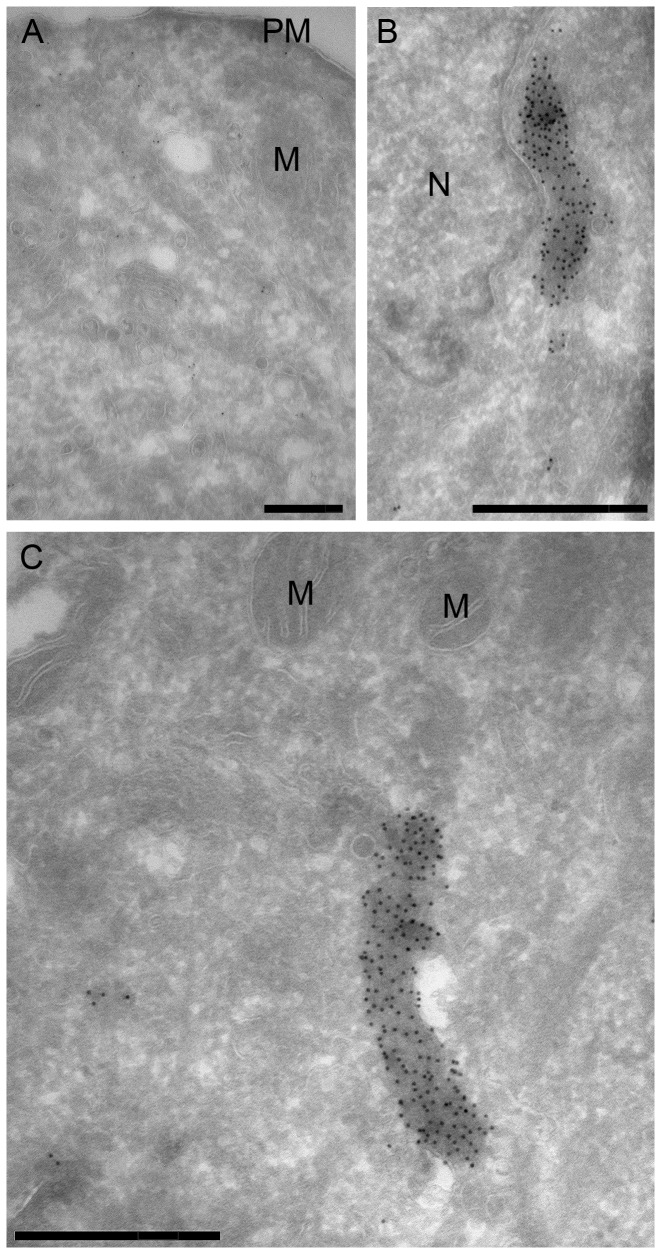
Immuno-EM of IMPDH localisation. Representative electron micrographs of CHO cells treated with (A) vehicle or (B and C) 2 µM MPA for 4 h. Cells were fixed, processed and labelled with anti-panIMPDH antibody and gold-labelled anti-mouse secondary antibody, as outlined in methods. Plasma membrane (PM), Nucleus (N) and mitochondria (M) are indicated. Scale bars = 0.5 µm.

**Figure 3:**
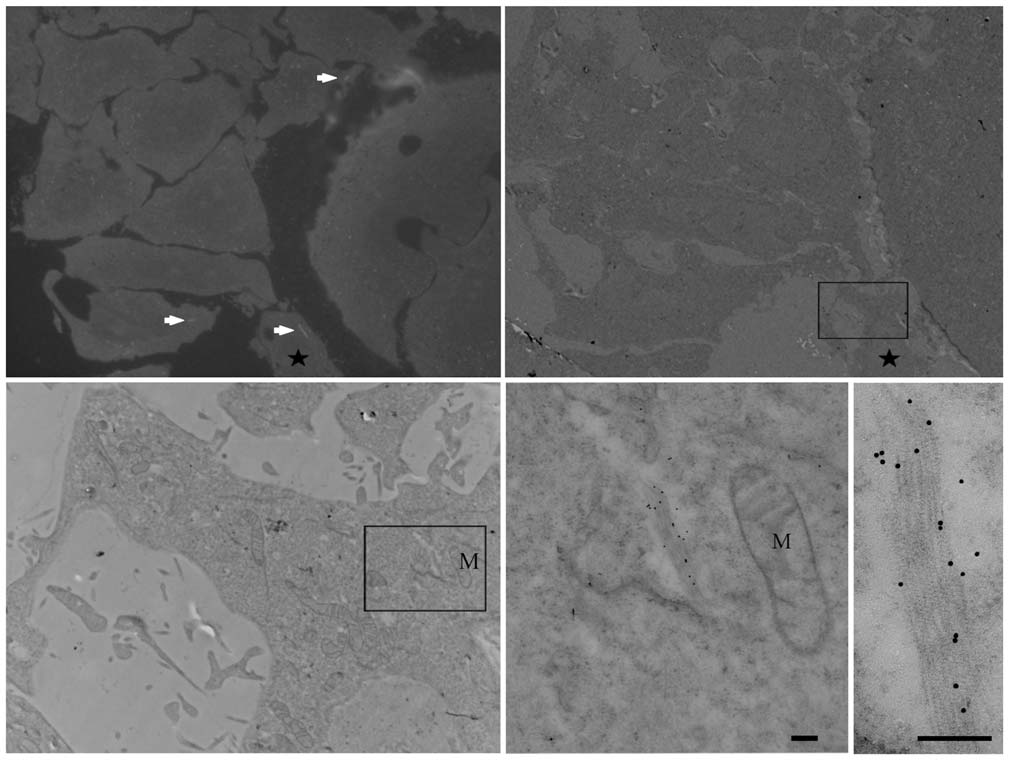
Analysis of IMPDH macrostructures in cryofixed material by correlative light and electron microscopy. HeLa cells selected for stable low expression of HA-IMPDH2-GFP were treated with 2 µM MPA for 4 h prior to high pressure freezing, freeze-substitution, and embedding in resin at low temperature. Semi-thin sections were viewed by fluorescence light microscopy to detect GFP-labelled macrostructures (arrows, upper left panel) and then ultrathin sections of the same areas were prepared for viewing in the TEM. Sections were immuno-gold labelled for detection of GFP. The panels show the same area (boxed) at increasing magnification to show the ultrastructure of the IMPDH macrostructures. Note the filamentous striated nature of the labelled elements in the cryofixed freeze substituted sample. Mitochondria (M) are indicated. Scale bars = 200 nm.

The ultrastructure of these macrostructures was further investigated by immuno-electron microscopy (EM) of ultra-thin cryosections of Chinese Hamster Ovary (CHO) cells treated with vehicle or MPA (Fig. 2). Consistent with the immunofluorescence, in control cells cryosections labelled for endogenous IMPDH showed sparse labelling distributed throughout the cytosol with no apparent association with any particular organelles (Fig. 2A). In contrast, after MPA treatment there was a dramatic labelling of elongated electron dense structures, often close to the nucleus (Fig. 2B and C) consistent with the formation of macrostructures observed by light microscopy (Fig. 1) and with previous studies [9], [23]. The unique morphology of these structures prompted us to further investigate their ultrastructure in cryofixed cells in the absence of primary fixation using a correlative light and electron microscopic approach. HeLa cells selected for stable low expression of a N-terminal HA-tagged, C-terminal GFP-tagged IMPDH2, HA-IMPDH2-GFP (Text S1 and Fig. S1), were grown on sapphire discs and then treated with MPA before rapid high pressure freezing and processing (Fig. 3). GFP-labelled macrostructures were visible in sections viewed by light microscopy allowing us to identify the same structures in parallel ultrathin sections viewed in the electron microscope and labelled with anti-GFP antibodies and 10 nm protein A-gold. The macrostructures were composed of linear elements up to a few microns in length comprising filamentous elements with regular striations (approximately 10 nm spacing).

### IMPDH clustering in live cells

**Figure 4:**
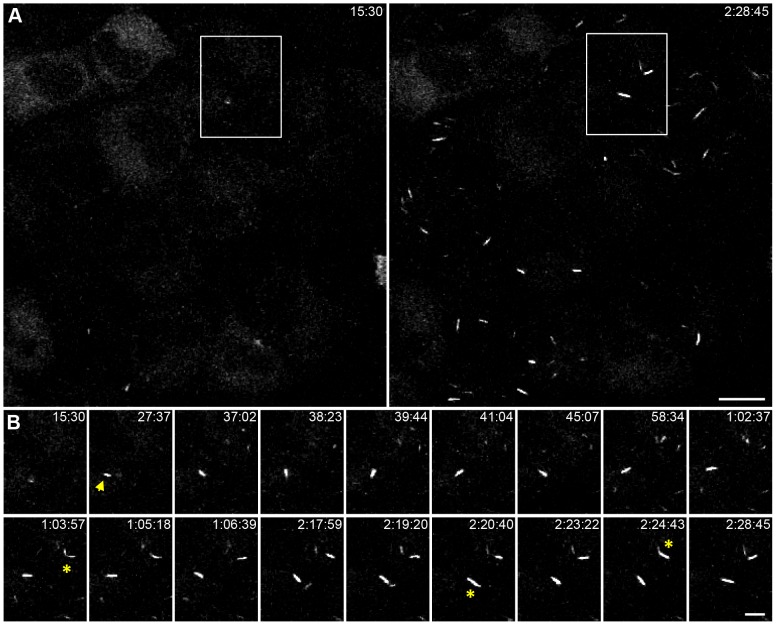
MPA induced clustering of HA-IMPDH2-GFP in live cells. Images are maximum intensity projections from confocal z-series of live HeLa HA-IMPDH2-GFP cells treated with 2 µM MPA. Selected frames, from Video S1, are presented with the time captured relative to the addition of MPA recorded at the top right (h∶mm∶ss). Data is representative of three independent experiments. (A) Field of cells with low HA-IMPDH2-GFP expression, which is initially diffuse throughout the cytosol and with time HA-IMPDH2-GFP clusters into spicules and macrostructures. Scale bar = 20 µm. (B) Selected frames within subset region shown in A, corresponds to Video S2, which highlight emergence of macrostructures, movement while undergoing a 180° clockwise rotation of in the X-Y plane (indicated by yellow arrow) and end-to-end fusion of spicules (indicated by yellow asterisk). Scale bar = 10 µm.

To investigate the dynamics of IMPDH redistribution we monitored clustering of GFP-tagged IMPDH2 fusion proteins using 4D time-lapse videomicroscopy. HeLa cells stably expressing low levels of HA-IMPDH2-GFP were treated with MPA (Fig. 4 and Video S1) or the GMP synthetase inhibitor, decoyinine (Fig. S2, Video S3 and S4), to induce macrostructure formation. Live cell imaging showed the time-dependent coalescence of HA-IMPDH2-GFP from a diffuse distribution throughout the cytoplasm into spicules, increasing in both number and intensity, and then into macrostructures. Moreover videomicroscopy revealed the processive nature of macrostructure formation which typically involves end-to-end fusion of spicules or less frequently lateral merging of spicules (Video S2). The length of the HA-IMPDH2-GFP macrostructures was typically shorter than those seen in cells expressing only endogenous or recombinant non-GFP-tagged IMPDH [9], although the temporal clustering of HA-IMPDH2-GFP was similar to that seen for endogenous IMPDH (Fig. S3). This probably reflects steric hindrance due to the large GFP moiety.

The above observations raised the question whether the cytoskeleton may play a role in coordinating the formation of macrostructures. Discombobulation of microfilament or microtubule networks, with cytochalasin D and nocodazole respectively, had no obvious effect on the ability of IMPDH to cluster upon decoyinine treatment (Fig. S4). While the cellular mechanisms driving macrostructure formation remains to be determined; these studies, combined with previous reports [9], [23], demonstrate that redistribution of IMPDH into macrostructures occurs in living cells in response to treatments that perturb intracellular nucleotide levels and importantly, do not represent an artefact of fixation or processing.

### Bateman domain alone does not confer IMPDH isoform clustering

**Figure 5:**
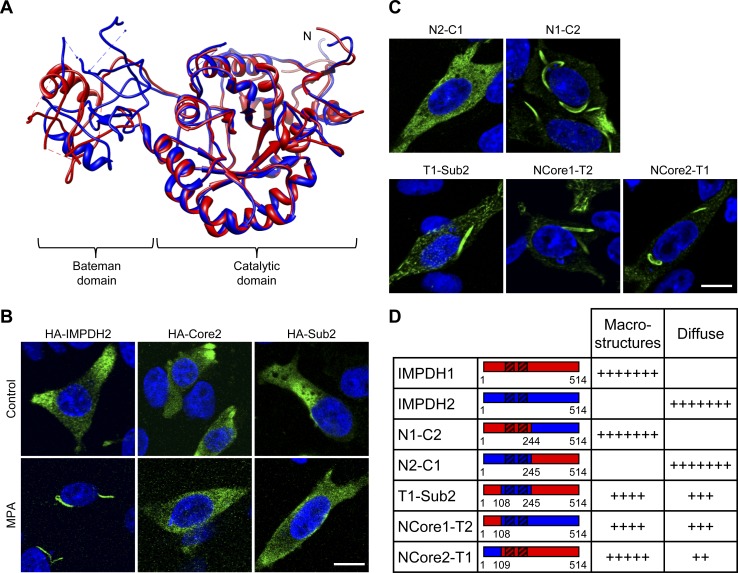
Investigating a role for the Bateman domain in IMPDH clustering. (A) Superimposed structure of IMPDH2 (1B3O; yellow) with IMPDH1 (1JCN; red). Ligands have been removed for clarity. N labels the N-terminus. (B) Micrographs of CHO cells transiently expressing HA-IMPDH proteins (as labelled), treated with vehicle (control) or 1 µM MPA for 4 h. Cells were fixed and labelled for HA (HA-IMPDH; green) and nuclei were counterstained with DAPI (blue). Representative of at least four independent experiments. (C) Representative micrographs of CHO cells transiently expressing IMPDH1/IMPDH2 chimera (as labelled). Cells were fixed and stained for HA (HA-IMPDH; green) and nuclei were counterstained with DAPI (blue). Scale bars = 10 µm. (D) Table shows qualitative scoring of chimera subcellular distribution pattern according to the similarity to IMPDH1 (macrostructure formation) or IMPDH2 (diffuse) distribution. Assignments were based on three independent experiments. Shown by the schematics are the IMPDH1 (red) and IMPDH2 (yellow) regions of chimera constructs, with internal numbering referring to IMPDH1 sequence boundary and the striped boxes indicating the CBS dimer.

We previously demonstrated that IMPDH1 has a higher propensity than IMPDH2 to cluster spontaneously into macrostructures [9]. Here we investigated whether a discrete region may underpin the divergence in IMPDH distribution. Alignment of the primary amino acid sequence of human IMPDH1 and IMPDH2 revealed 25% sequence variability within the Bateman domain containing “sub” domain (amino acids 99–244), with CBS1 having 27% variability compared to 8% in CBS2. Whereas there was only 12% sequence variation in the catalytic “core” domain (amino acids 1–108+245–514). Superimposition of solved crystal structures [24], [25] further highlighted the conservation in tertiary structure of the catalytic domain (Fig. 5A). Together, this advocated the Bateman domain as the candidate region underpinning the intrinsic differences between the IMPDH isoforms.

To investigate the requirement of the Bateman domain in clustering the localisation of HA-IMPDH2 sub and core constructs, based on those of Nimmesgern *et al.*, (1999) [17], were examined by indirect immunofluorescence (Fig. 5B). Transiently expressed full-length HA-IMPDH2 redistributed into macrostructures following MPA treatment [9]. In contrast, the sub and core proteins displayed a diffuse cytoplasmic localisation in both control and MPA treated cells. This suggests the intact enzyme is required for redistribution, however, these results do not establish whether the Bateman domain plays a direct structural role and/or a regulatory role in clustering.

Next, three generations of IMPDH1/IMPDH2 chimera constructs were designed to assess if the Bateman domain confers the difference in spontaneous clustering. The HA-IMPDH chimeras expressed as full-length active enzymes (Fig. S5), indicating the chimeras were of sound structural and functional integrity. Indirect immunofluorescence was employed to determine the subcellular distribution of the chimeras (Fig. 5C) and this was also qualitatively compared to the wild-type isoforms based on propensity to spontaneously form macrostructures, number of clusters per cell, localisation and form of the macrostructures (Fig. 5D). The first generation chimeras, N1-C2 and N2-C1, had subcellular localisation patterns akin to IMPDH1 and IMPDH2 respectively, demonstrating that the N-terminal 244 amino acids determines the propensity to spontaneously form macrostructures. Perhaps surprisingly, the T1-Sub2 chimera, with an IMPDH2 Bateman domain, clustered spontaneously albeit to a lesser extent than IMPDH1 (macrostructures were typically smaller and accompanied by spicules). Intriguingly, chimeras NCore1-T2 and NCore2-T1, which were generated by exchanging amino acids 1–108 between isoforms, both displayed an intermediate propensity to form spontaneous macrostructures. Together, this suggested a role for communication between the Bateman domain and catalytic domain which is likely to be integral for IMPDH clustering.

### IMPDH isoforms bind adenosine nucleotides in an isoform-specific manner

Bateman domains serve as allosteric regulatory modules in a range of proteins with nucleotide binding to the Bateman domain promoting this function [16], [22]. Nucleotide binding studies, to date, have been limited to IMPDH2 and compared only ATP and GTP. We next assessed the purine nucleotide binding characteristics of purified, recombinant His-IMPDH proteins using a protease protection assay. The protease protection assay was based on the partial proteolysis assay previously used to investigate ligand induced conformational changes in the active site of hamster IMPDH2 [26], which differs from human IMPDH2 by only six amino acids [1].

**Figure 6:**
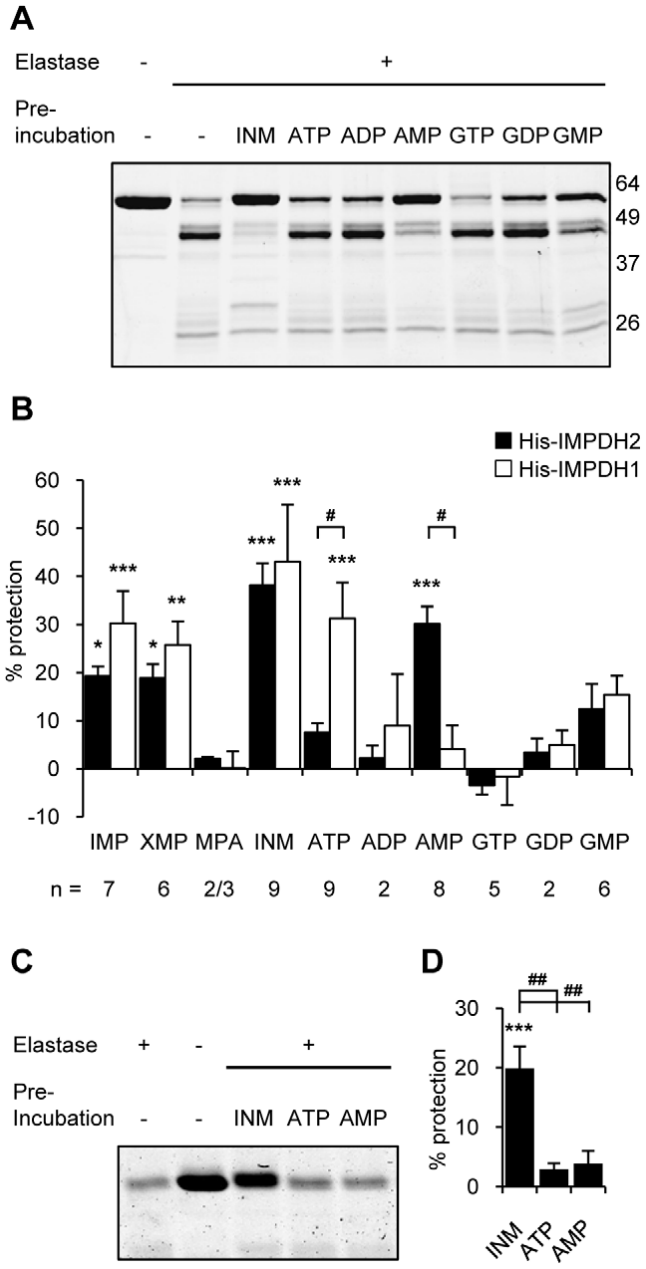
Nucleotides protect IMPDH in an isoform-specific manner via the Bateman domain. (A) Representative Coomassie stained SDS-PAGE gel of a protease protection assay experiment, performed as outline in methods, with His-IMPDH2. INM stands for IMP, NAD and MPA. Molecular weight marker sizes are indicated. (B) Quantitation of remaining full-length protein from protease protection assay with His-IMPDH2 and His-IMPDH1 presented as percent protection. Shown is the mean ± SEM and the number of experiments (n) is indicated below the graph. (*p<0.05, **p<0.01, ***p<0.001 *cf.* the control (digested), # *p*<0.01 IMPDH1 *cf.* IMPDH2). (C) Representative gel of a protease protection assay experiment with His-Core2 protein. (D) Quantitated results as per B. Shown is the mean ± SEM from five independent experiments. (***p<0.001 *cf.* the control (digested), ## *p*<0.001 IMP, NAD and MPA *cf.* adenosine nucleotides).

We observed, in the absence of substrate or substrates and inhibitor that both human IMPDH isoforms, like hamster IMPDH, undergo time-dependent proteolysis with the protease-sensitive region mapping within the catalytic domain (Text S2 and Fig. S6). Both IMPDH1 and IMPDH2 proteins showed reduced proteolysis with pre-incubation of IMP, although pre-incubation of IMP, NAD and MPA, which results in the formation of a complex between a covalent enzyme-substrate intermediate and MPA [20], [27], afforded greater protection from proteolysis and this was to a similar extent with both isoforms (Fig. 6A and B). These results suggest that both human IMPDH isoforms exhibit conformational changes upon substrate/inhibitor binding in the active site.

**Figure 7:**
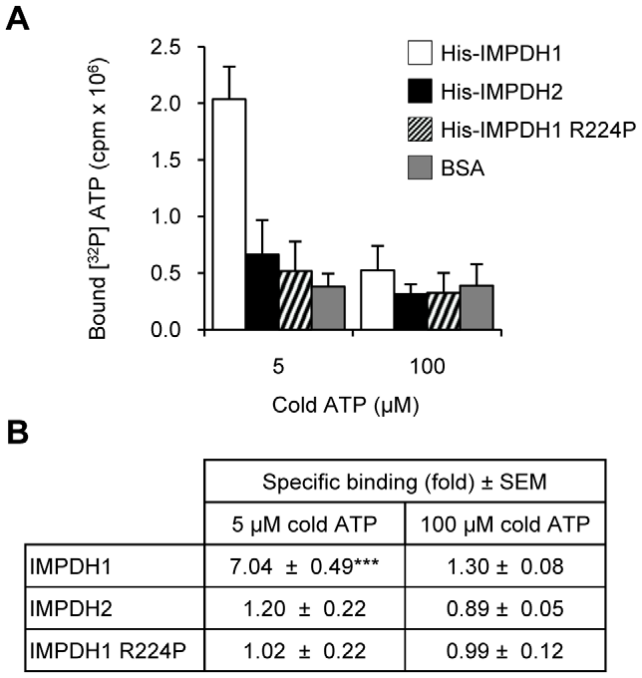
IMPDH1 directly binds ATP. (A) Representative ATP binding experiment with His-IMPDH proteins, shows [^32^P] ATP bound to His-IMPDH1 only in the presence of 5 µM cold ATP. Data represents mean counts ± SD. (B) Specific binding was determined by expressing the counts per reaction as a fold over the non-specific counts present in the BSA sample. Shown is the mean specific binding ± SEM (fold over non-specific binding) from three to six independent experiments (***p<0.001 compared to the control).

We then reasoned that purine nucleotide binding to the Bateman domain of IMPDH may elicit conformational changes within IMPDH leading to altered protease accessibility to the cleavage site. A significant and striking difference between the isoforms was observed with the pre-incubation of AMP and ATP. AMP resulted in significant protection of IMPDH2 (30%; Fig. 6A and B). In contrast to IMPDH2, negligible protection of IMPDH1 was afforded by AMP. However, IMPDH1 protein was significantly protected from proteolysis by ATP (31%; Fig. 6B) whilst this effect was not seen for IMPDH2. This observed protection of IMPDH1 by ATP and IMPDH2 by AMP was also shown to be dose-dependent (Fig. S7). No significant protection of either IMPDH2 or IMPDH1 was observed with equimolar concentrations of the other purine nucleotides. Specific ATP binding to IMPDH1, but not IMPDH2, was supported by a direct filter binding assay (Fig. 7A and B). These results suggest that IMPDH exhibits isoform specificity for ATP and AMP, which may elicit conformational changes within the enzyme, highlighting further differences between the isoforms.

### Bateman domain mediates nucleotide protection

GMP and, to a lesser extent, AMP have been reported to act as competitive inhibitors of IMPDH [28], [29]. Hence, to ascertain that the protection afforded by AMP was modulated via the Bateman domain, and not via binding to the active site, the protease protection assay was performed with a purified, recombinant His-IMPDH2 core protein (Fig. 6C and D). As expected, the core protein was significantly protected by pre-incubation with IMP, NAD and MPA. In contrast to the IMPDH2 protein, AMP did not confer protection of the core protein. This data suggests that the Bateman domain is integral for the protection afforded by nucleotides and supports the hypothesis that nucleotides bind the Bateman domain.

### The R224P mutation disrupts ATP binding and IMPDH1 subcellular distribution

**Figure 8:**
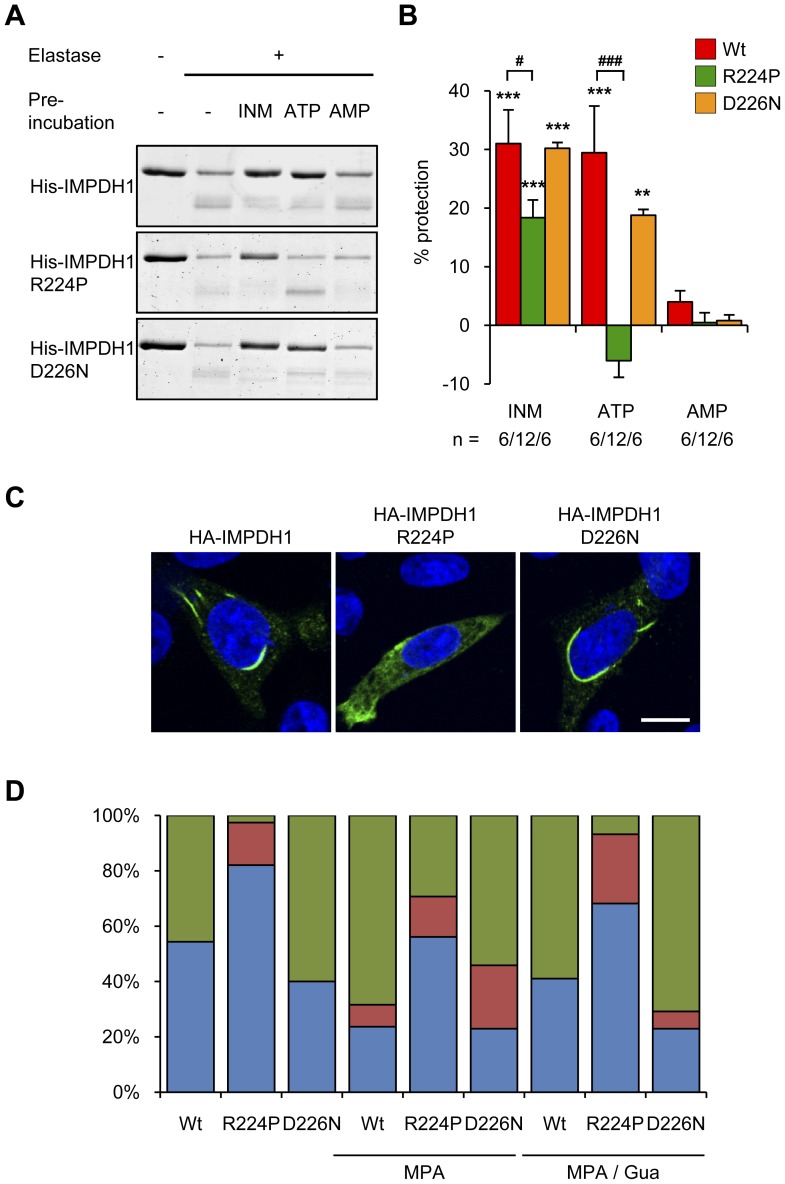
R224P mutation affects ATP mediated protease protection and affects spontaneous clustering. (A) Representative Coomassie stained gel of a protease protection assay with His-IMPDH1 proteins. INM stands for IMP, NAD and MPA. (B) Quantitation of remaining full-length protein from protease protection assay presented as percent protection. Shown is the mean ± SEM and the number of experiments (n) is indicated below the graph. (*p<0.05, **p<0.01, ***p<0.001 *cf.* the control (digested), # *p*<0.01 *cf.* IMPDH1. (C) Representative micrographs of CHO cells transiently expressing HA-IMPDH1, HA-IMPDH1 R224P or HA-IMPDH1 D226N. Cells were fixed and stained for HA (HA-IMPDH; green) and nuclei were counterstained with DAPI (blue). Representative of at least four independent experiments. Scale bar = 10 µm. (D) CHO cells transiently expressing HA-IMPDH proteins, as indicated, were treated with either vehicle, 2 µM MPA for 4 h or 2 µM MPA for 4 h and supplemented with 100 µM guanosine for the final 2 h. Cells were fixed and stained with anti-HA antibody. From random fields, 50–80 labelled healthy cells were counted, in a blinded manner, and the classification of the subcellular distribution of the protein classified as diffuse (blue), in spicules (red) or macrostructures (green). Representative of two independent experiments.

The effects of the most-common RP-causing mutations on the ATP-mediated protection of IMPDH1 were examined (Fig. 8A and B). Consistent with wild-type protein, pre-incubation of the purified, recombinant mutant proteins with IMP, NAD and MPA significantly protected the enzymes from proteolysis, although protection of the R224P mutant was significantly less than wild-type. The R224P protein was not protected by ATP. Furthermore, no ATP binding with the R224P mutant was confirmed with a direct filter binding assay (Fig. 7A and B). In contrast, the D226N mutant was significantly protected from protease digestion by ATP, albeit qualitatively less than wild-type. Similar to IMPDH1, neither mutant protein was protected by pre-incubation with AMP.

This striking affect of the R224P, and not the D226N, mutation on IMPDH1 properties correlated with an altered subcellular distribution of this mutant protein (Fig. 8C and D). Unlike wild-type, the R224P mutant had a very low tendency to spontaneously cluster into macrostructures (<3%). HA-IMPDH1 R224P predominantly displayed a diffuse cytoplasmic pattern with spicules being detected in 5–15% of cells. In contrast, the D226N mutant had a high propensity (60–75% cells) to spontaneously cluster into macrostructures. Treatment with MPA promoted clustering of R224P and D226N mutant proteins, although to a lesser extent in cells expressing the R224P mutant compared with either the wild-type or D226N mutant proteins, which were similar (Fig. 7C and D).

### AMP and ATP are not allosteric activators of IMPDH

**Figure 9:**
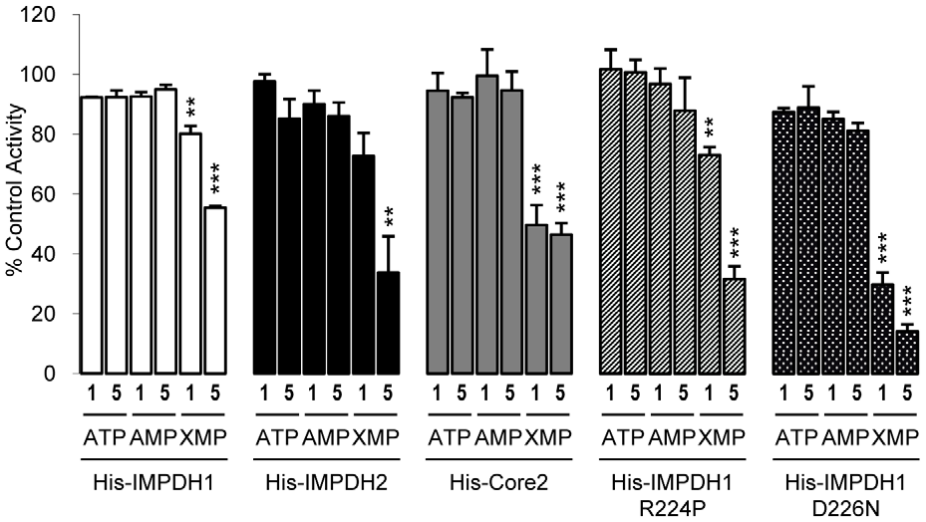
IMPDH activity in the presence of nucleotides. Activity of purified His-IMPDH proteins following 20 min pre-incubation with 1 mM or 5 mM nucleotides (ATP, AMP, XMP) and normalised to control, no addition, activity. Shown is the mean ± SEM (n = 3, **p<0.01, ***p<0.001 *cf.* to the control activity).

Finally we investigated the catalytic activity of the purified, recombinant His-IMPDH proteins in the presence of ATP, AMP or XMP (Fig. 9). Compared with the inhibitory effects of XMP, no significant effects on activity were observed with either ATP or AMP. Importantly, in light of a previous report [22], there was no evidence of an increase in activity of any of the IMPDH proteins upon incubation with ATP. Intriguingly, the D226N mutant showed a substantial 75–85% reduction in activity with XMP (p<0.001 compared to wild-type) which suggests that this disease-causing mutation may increase sensitivity to product inhibition.

## Discussion

The current studies were prompted by the observation that IMPDH1 and IMPDH2 may be distinguished by their propensity to spontaneously cluster into macrostructures [9]. Here we report isoform-specific differences in nucleotide binding, via a mechanism involving the Bateman domain, which results in conformational changes communicated between the catalytic domain and the Bateman domain, consistent with a role for both domains in determining the subcellular distribution of IMPDH.

Our microscopy analyses further defined the ultra-structure of IMPDH macrostructures and provided insight into the dynamics of clustering, but the functional significance of IMPDH clustering remains an enigma. Our previous study showed the enzymes upstream (aminoimidazolecarboxamide ribonucleotide formyltransferase/IMP cyclohydrolase (ATIC)) and downstream (GMP synthetase) of IMPDH in the *de novo* synthesis pathway did not redistribute with IMPDH, ruling out a role for substrate channelling [9]. Interestingly, An and colleagues described co-localisation of enzymes situated upstream of IMPDH in the *de novo* pathway of purine biosynthesis, including ATIC, into rounded punctate structures in response to changes in purine levels [30]. Assembly of these “purinosome” complexes appears dependent on casein kinase II and, in contrast to IMPDH macrostructures, are stabilised by microtubules [31]. Under conditions ascribed to result in purinosome formation [30] we observed modest redistribution of IMPDH into small macrostructures that did not co-localise with a key component of the purinosomes, formylglycinamidine-ribonucleotide synthetase (FGAMS)-OFP [30], or with ATIC (ECT and JPW unpublished data). Collectively these observations indicate that IMPDH macrostructures are distinct from purinosomes. One possibility is that this alternate distribution may facilitate preferential channelling of IMP into the biosynthetic pathway of adenine nucleotides rather than guanine nucleotides.

Emerging evidence suggests protein clustering may be a general response to metabolic challenge. Recently, 180 yeast proteins, including an IMPDH homolog, were shown to cluster reversibly to punctate “foci” upon induction of quiescence by nutrient starvation [32]. A subsequent screen identified nine additional GFP-tagged proteins which self-assembled into filamentous structures, reminiscent of macrostructures, in response to distinct environmental conditions [33]. Although the yeast IMPDH homolog IMD was not found to cluster it is possible that the GFP-tag prevented this, since it was necessary for us to express human IMPDH2-GFP at low levels in order to observe clustering. Also of note was the assembly of the cytidine triphosphate synthase (CTPS) homolog Ura7p into filaments following glucose deprivation or treatment with sodium azide, suggesting Ura7p distribution may also be sensitive to cellular energy status. Interestingly, a recent study by Carcamo and colleagues reported that CTPS1 was present in macrostructures and that inhibition of CTPS was sufficient to promote macrostructure formation [34]. These observations are largely consistent with those from the Mitchell lab [23] and our own ([9] and the current study) and suggest that CTPS1 is present in macrostructures. However, it is noteworthy that *in vitro* studies, using purified IMPDH, demonstrate that IMPDH is able to form macrostructures in the absence of additional proteins [23]. Moreover, biochemical analyses have failed to identify interacting partners for IMPDH [23], [35](ECT and JPW unpublished) suggesting any interaction between IMPDH2 and CTPS1 may be relatively labile. Nevertheless, further investigations of macrostructures should include examination of the potential role and contribution of CTPS1.

Our studies suggested an unappreciated role for communication between the Bateman and catalytic domains which are likely to be integral to the specific regulatory properties of IMPDH isoforms. The protease protection assay provided insight into the nucleotide binding specificity of the isoforms under conditions that allowed us to compare the effects of all purine nucleotides in parallel on both isoforms. Interestingly, previous studies suggested IMPDH2 could bind GTP [23] and ATP [22]. Whilst the latter study also reported that ATP binding increased activity four-fold this finding has not been recapitulated here or by others [12], [21], [23]. Although we cannot exclude the possibility that IMPDH proteins may bind GTP [23], unlike adenosine nucleotide binding to IMPDH, our findings imply that it occurs without concurrent conformational changes. Recent structural and biochemical studies of Bateman domain containing proteins including MJ0100 [36], Cystathionine β-Synthase [37] and AMPKγ [15], [38] suggest that such ligand-induced conformational changes represent an important allosteric regulatory mechanism.

The significance of the isoform specificity of IMPDH nucleotide binding remains to be elucidated, however, an attractive possibility is that it may reflect an adaptation to different demands of specific cell types. Our attempt to characterise and compare the ‘energy sensing ability’ of the human IMPDH isoforms *in vivo*, in *gua*B null *E.coli*, proved unsuccessful since only human IMPDH2, but not IMPDH1, was able to complement growth (ECT and JPW unpublished data). It is intriguing that ADP was without significant affect on the proteolysis profile of either isoform, particularly IMPDH1, since it would be anticipated that the binding pocket would be sufficiently large to accommodate ADP. Whilst our observations of IMPDH binding adenosine nucleotides are in accordance with biochemical and structural studies of other Bateman domain containing proteins [22], [39], [40], [41], [42] recent reports suggest that ADP binding to the regulatory γ subunit of AMPK may represent a key event in the allosteric regulation of AMPK activity [15], [38], [43]. Further, detailed investigations into the specificity of nucleotide binding of the IMPDH isoforms are warranted. Moreover, detailed analysis of the kinetics of activation of IMPDH1 and IMPDH2 by nucleotides, including AMP and ATP, may provide additional and complementary insights into the molecular mechanisms governing the allosteric regulation of the IMPDH isoforms. However, such investigations were beyond the scope of the current work.

The occurrence of disease-causing mutations in and around the Bateman domain of IMPDH1, which have no effect on enzymatic activity [10], [11], [12], together with our findings from the chimeras suggests a physiologically important role of a regulatory region outside the catalytic site of the enzyme. Currently, there is no consensus as to the molecular effects yielded by RP-causing mutations in IMPDH1 and intriguingly, the reported properties are divergent between the R224P and D226N mutants [10], [11], [12], [44], [45], [46]. In the current study, the RP-causing mutation, R224P, abolished ATP binding and this correlated with a reduced propensity to cluster. In contrast the D226N mutant protein had properties similar to wild-type, although there was a trend towards reduced ATP binding. Interestingly, the D226N mutant showed increased sensitivity to inhibition by XMP, which raises the intriguing possibility that this mutation may elicit pathogenicity by enhancing the product-inhibition of IMPDH. In developmental terms, it is likely that any effects of dysregulation are initially masked by IMPDH2 being the predominant isoform in the developing retina with expression of IMPDH1 proteins dominating only in the mature retina [9]. Future studies will be required to examine the effect of these disease-causing mutations in the context of the major retinal variants (type Iα and γ). Moreover, determination of the functional significance of wild-type protein binding ATP will provide insight into the consequence of the properties disrupted by the R224P mutation.

Recently, the Bateman domain of IMPDH has been revealed to be a negative transregulator of adenosine nucleotide synthesis in *E.coli* [21], [47]. *E.coli* that were recombineered to express IMPDH lacking a Bateman domain were sensitive to adenosine or inosine induced growth arrest. This corresponded with an increase in the adenosine nucleotide pool, resulting in allosteric inhibition of PRPP synthase, reduced PRPP and subsequent pyrimidine starvation [47]. While the molecular mechanism remains to be elucidated, Pimkin *et al.*, (2009) [47] suggested that this may occur via an interaction with and inhibition of adenylosuccinate synthetase. This raises the intriguing possibility that the nucleotide-bound-state of IMPDH may contribute to the regulation of adenosine nucleotide synthesis. Congruent to this, we propose that IMPDH clustering may confer another level of regulation that contributes to modulating a function of IMPDH outside of, although perhaps linked to, IMP catalysis. Indeed, the observations of this study are complementary with the suggestions that IMPDH has additional unappreciated “moonlighting” functions which are distinct from its role in guanine nucleotide biosynthesis. IMPDH has been implicated in lipid accumulation [35], [48], proposed to be involved in RNA metabolism, due to an association of nucleic acids via the Bateman domain [12], [44], [45], [46] and most recently described as a DNA-binding transcriptional repressor [49]. A common feature of all these proposed functions for the Bateman domain of IMPDH is that it is likely to either regulate IMPDH and/or be implicated in regulation afforded by IMPDH. Furthermore, it is likely that additional levels of regulation, such as phosphorylation [35], [50], [51], contribute to the complexity of IMPDH modulation and allow for adaptation to the intracellular environment.

In summary, in the present study we have demonstrated that the nucleotide binding properties of the IMPDH isoforms differ. Together with the striking differences between the propensities to cluster into macrostructures these findings emphasise that IMPDH1 and IMPDH2 have distinct properties. Moreover, a disease causing mutation in IMPDH1, R224P, altered these distinguishing properties. Collectively, these results raise the possibility that the nucleotide sensing properties of the Bateman domain in IMPDH serve to regulate IMPDH and co-ordinate nucleotide homeostasis, thereby giving rise to cellular plasticity in an isoform-specific manner to meet the requirements of the cellular environment.

## Materials and Methods

### Reagents and Materials

Reagents were from Sigma–Aldrich (Castle Hill, NSW, Australia) unless otherwise stated. Tissue culture reagents and foetal bovine serum were from Invitrogen (Mount Waverley, VIC, Australia) and Bovogen Biologicals (Essendon, VIC, Australia) respectively. The anti-panIMPDH antibody was a kind gift from Frank Collart [52], the anti-HA antibody was from Covance (Berkley, CA, USA), the anti-tubulin antibody from Abcam (Cambridge, UK), the anti-GFP antibody [53] and isoform-specific IMPDH antibodies [9] were generated as previously described. All secondary antibodies were from Molecular Probes (Eugene, OR, USA).

### Generation of IMPDH constructs

To yield pmEGFP-C1 HA-IMPDH2-GFP, HA-IMPDH2 cDNA [35] was amplified by PCR with forward 5′-GGTGGTGCTAGCGCCACCATGTACCCATACGATGTGCCAGATTACGCT-3′ and reverse 5′- GGTGGCGACCGGTCCACCAGAACCACCTGCACCAGATGCACCTGTTCCGAAAAGCCGCTTCTCATACG-3′ primers, which was inserted into pmEGFP-C1 (Clontech, Mountain View, CA, USA) on NheI/AgeI sites. IMPDH chimeras and the truncated core domain constructs were cloned with an N-terminal HA-tag using a three-step PCR method (see Table S1 for primers) employed by Nimmesgern *et al.*, (1999) [17]. Firstly, two distinct PCR products, A and B, were generated using template and primer pairs detailed in Table S1. In the second round, PCR products A and B became the template for amplification with the forward primer of PCR A and reverse primer of PCR B resulting in a chimeric full-length product. This amplicon was shuttled into Blunt II TOPO (Invitrogen) prior to subcloning on HindIII/NotI site into pcDNA5/FRT/TO (Invitrogen). A hexa-histidine (His)-tag was inserted at the 5′ end of human IMPDH1 or IMPDH2 cDNA [35] by PCR prior to cloning into pET20b (+) (Novagen, Madison, WI, USA). QuikChange site-directed mutagenesis kit (Stragene, La Jolla, CA, USA) was used to introduce point mutations, CGC to CCC (R224P) and GAC to AAC (D226N).

### Cell Culture

Chinese Hamster Ovary (CHO) cells and HeLa cells were cultured in complete F12 HAMs and Dulbecco's Modified Eagle's medium respectively, supplemented with 10% FBS and 2 mM L-glutamine. Cells were transfected and treated as previously described [9]. HeLa cells stably expressing HA-IMPDH2-GFP were initially selected, and subsequently maintained, with geneticin (600 µg/ml) added to the media 24 h post transfection with the pmEGFP-C1 HA-IMPDH2-GFP plasmid. A population of cells with a low fluorescence, due to low expression of HA-IMPDH2-GFP, were further selected by fluorescence activated cell sorted analysis. The resulting heterogeneous stable population of cells expressed HA-IMPDH2-GFP at approximately 10% of the level of endogenous IMPDH. Cells were treated with either vehicle, 2 µM MPA for either 4 h or as indicated or 2 µM MPA for 4 h and supplemented with 100 µM guanosine for the final 2 h.

### Immunofluorescence and time-lapse videomicroscopy

Indirect immunofluorescence microscopy was performed as previously described [9] with cells being imaged on a LSM510 META confocal microscope at 100× magnification (Carl Zeiss MicroImaging, Jena, Germany). For 3D time-lapse (4D) videomicroscopy, HeLa HA-IMPDH2-GFP cells were cultured on 24 mm glass bottomed dishes (Proscitech, Qld, Australia). Cells were washed with PBS and complete F12 HAMS prior to replacing with complete F12 HAMS containing the inhibitors. Cells were then imaged, on a pre-heated (37°C) stage top insert with 5% humidified CO_2_ circulating, through a C-Apochromat 40×/1.20 W Korr UV-VIS-IR M27 objective using 4–6% 488 nm laser intensity on a LSM510 META confocal microscope (Zeiss). Confocal Z-series (0.9–1.1 µm increments) were acquired over time using the LSM software (Zeiss) and collected images were processed, analysed and movies constructed using Image J v1.41 software (NIH). All images and movies are maximum intensity projections of the 3D image.

### Electron microscopy and correlative light and electron microscopy

CHO cells were fixed with 0.1% glutaraldehyde/4% PFA and processed for EM as previously described [54]. Sections were labelled with antibodies to IMPDH followed by 10 nm protein A-gold. For cryofixation and correlative light and electron microscopy, HeLa cells selected for stable low expression of HA-IMPDH2-GFP were treated with MPA and then high pressure frozen, freeze substituted and embedded in HM20 resin at low temperature as described previously [55] with modifications to the Lowicryl HM20 infiltration which was shortened to one day (50%, 75% and 100% for 1 h each followed by two 12 h 100% infiltrations all at −50°C). Sections were labelled with antibodies to GFP followed by 10 nm protein A-gold.

### *In silico* analysis

UCSF Chimera (version 1.3; [56]) was used to coordinate superimposition of protein data bank files for human IMPDH2 (1B3O; [24]) and IMPDH1 (1JCN; [25]), utilising the default parameters of the matchmaker function, and for the structure visualisation.

### Protein purification

*Escherichia coli* BL21 (DE3) transformed with pET20b constructs were induced at room temperature (RT) for 12–14 h by addition of isopropyl-beta-D-thiogalactopyranoside (1 mM). Cell pellets were resuspended in either binding buffer 1 (50 mM Tris pH 8.0, 100 mM KCl, 30 mM imidazole, 1.5 M urea, 10 mM 2-mercaptoethanol) for IMPDH1 proteins and the core protein or binding buffer 2 (50 mM Tris pH 6.8, 500 mM KCl, 30 mM imidazole) for IMPDH2, containing protease inhibitors (1 µg/ml leupeptin, 1 µg/ml pepstatin, 1 µg/ml antipain, 250 µM benzamidine and 3 mM AEBSF). Lysates were sonicated, centrifuged at 17000× *g* for 30 min at 4°C and His-IMPDH proteins purified on a talon affinity resin (Clontech) or nickel-nitriloacetic acid column (Invitrogen) according to the manufacturer's instructions. Protein was eluted with appropriate binding buffer containing 250 mM imidazole and dialysed into activity assay buffer (100 mM KCl, 100 mM Tris-HCl pH 8.0, 1 mM DTT) with glycerol added to a final concentration of 20% for storage.

### Protease protection assay

Purified His-IMPDH protein (0.9 µM) was incubated for 20 min at RT with 1 mM nucleotides or 1 mM MPA with 1 mM IMP and NAD in activity assay buffer prior to addition of 20 µg/ml elastase for a further 20 min. Reactions were ceased by addition of Laemmli SDS-PAGE sample buffer and heat denaturation, then analysed by SDS-PAGE. Protein bands were visualised with coomassie staining and the full-length (intact) protein quantitated using the LI-COR Odyssey Infrared Imaging System. Percentage protection was calculated using the following formula: % protection = ((full-length protein remaining after digestion in sample/undigested protein) – (full-length protein remaining with elastase only digestion/undigested protein))×100%.

### [^32^P] ATP filter binding assay

The ATP binding assay was based on those previously described [22], [23], [57]. In brief, purified His-IMPDH protein (0.9 µM), or BSA (used as a negative control), was mixed with 1 µM [α-^32^P] ATP (800 Ci/mmol; Perkin Elmer, Waltham, MA, USA) and cold ATP (5 µM or 100 µM) for 20 min at RT in binding buffer (50 µl total reaction −100 mM KCl, 100 mM Tris-HCl pH 8.0, 1 mM DTT and 2.5 µM BSA) in duplicate. Reactions were terminated by rapid filtration, loading 15 µl onto 3× pre-equilibrated (in binding buffer) MF filter membrane discs (Millipore, Billerica, MA, USA) under vacuum. Filters were washed rapidly (<20 s) with 4×150 µl of ice-cold binding buffer, dried and radioactivity bound to filters measured by liquid scintillation counting.

### Measurement of IMPDH activity

The IMPDH activity assay of purified protein was based on that described for crude lysates [9] and the protease protection assay. In duplicate wells of a 384-well plate, purified His-IMPDH proteins (0.9 µM) were incubated in activity assay buffer containing 3 mM EDTA to a final volume of 38 µl with 1 mM or 5 mM nucleotides (ATP, AMP, XMP) and 0.5 mM NAD for 20 min at RT. IMP (0.5 mM) was added and the enzymatic activity was measured at 37°C by monitoring NADH production (*A*_340_ nm). Background measurements were determined with a parallel sample without IMP. IMPDH activity reflects the rate of change during a linear 15 min period.

### Statistics

Statistical analysis was performed using GraphPad Prism version 5.00 for Windows (GraphPad Software, San Diego, CA, USA). Data were analysed using ANOVA and a Bonferroni post-hoc test to compare the means between different treatments.

## Supporting Information

Text S1 **Supporting information for HA-IMPDH2-GFP fusion protein.**
(DOCX)

Text S2 **Supporting information mapping the elastase cleavage site of human IMPDH.**
(DOCX)

Figure S1 **Characterisation of HA-IMPDH2-GFP fusion protein.** (A) Cartoon of HA-IMPDH2-GFP fusion protein with the linker sequence indicated between IMPDH and GFP (green box). Yellow box represents the HA-tag, maroon boxes indicates the CBS domains and the catalytic domain is shown in blue. (B) Lysates (20 µg) of HeLa cells transiently expressing either GFP (lane 1), HA-IMPDH type II (lane 2) or HA-IMPDH2-GFP (lane 3) or the stable HeLa HA-IMPDH2-GFP cells (lane 4) were analysed by SDS-PAGE and western blotting. Membrane was probed with anti-GFP antibody (red) and anti-panIMPDH antibody (green). Asterisks indicate non-specific bands. (C) IMPDH activity was determined in cell lysates of HeLa cells transiently expressing HA-IMPDH2-GFP or HA-IMPDH2 or GFP alone, to control for endogenous IMPDH activity, as previously described [9]. Activity was normalised to exogenous protein expression, as determined by western blot, and the values expressed in terms of HA-IMPDH2 activity. Data represents the mean ± SD of two independent experiments. (D) Representative epifluorescence micrographs of HeLa cells transiently expressing HA-IMPDH2-GFP (green). Cells were incubated with vehicle (control) or with 2 µM MPA for 4 h. Cells were fixed, permeabilised and labelled with the anti-panIMPDH antibody and Alexa-594 secondary antibody (red) and nuclei were counterstained with DAPI (blue). Images are representative of at least three independent experiments. Scale bar = 20 µm.
(TIF)

Figure S2 **Decoyinine induced clustering of HA-IMPDH2-GFP in live cells.** Images are maximum intensity projections from confocal z-series of live HeLa HA-IMPDH2-GFP cells treated with 2 mM decoyinine. Selected frames, from Video S3, are presented with the time captured relative to the addition of decoyinine recorded at the top right (h∶mm∶ss). Data is representative of three independent experiments. Scale bar = 10 µm. (A) Field of cells with low HA-IMPDH2-GFP expression, which is initially diffuse throughout the cytosol and with increasing time HA-IMPDH2-GFP clusters into spicules and macrostructures. (B) Selected frames within subset regions shown in A, corresponds to Video S4, which highlight the emergence and lateral fusion (indicated by yellow asterisk) of a HA-IMPDH2-GFP macrostructure.
(TIF)

Figure S3 **Time course of endogenous IMPDH clustering.** Micrographs of CHO cells treated with vehicle (control) or 2 µM MPA for the times indicated. Cells were fixed and labelled for endogenous IMPDH (green) and nuclei were counterstained with DAPI (blue). Representative of at least three independent experiments.
(TIF)

Figure S4 **Microfilament or Microtubules alone are not required for formation of IMPDH macrostructures.** Representative confocal micrographs of HeLa HA-IMPDH2-GFP cells. (A) Cells were treated with either vehicle or 4 µM cytochalasin D (CD), to discombobulate microfilaments, for 1 h 15 min prior to either fixation (top panel) or addition of 2 mM decoyinine (DCN) for an additional 3 h 30 min (bottom panel). Cells were fixed, permeabilised and labelled with the anti-panIMPDH antibody (green), filamentous actin was stained with TRITC-phalloidin (red) and nuclei were counterstained with DAPI (blue). Images are representative of at least three independent experiments. Scale bar = 20 µm. (B) Cells were treated with either vehicle or 20 µM nocodazole (Noc), to destabilise microtubules, for 1 h 15 min prior to either fixation or addition of 2 mM decoyinine (DCN) for an additional 3 h 50 min. Cells were fixed, permeablised and labelled with the anti-tubulin antibody (left hand panel; red) or anti-panIMPDH antibody (right hand panel; red) and nuclei were counterstained with DAPI (blue). In green is the low fluorescence of the HA-IMPDH2-GFP. Images are representative of at least two independent experiments. Scale bar = 20 µm.
(TIF)

Figure S5 **Expression and activity of IMPDH1/IMPDH2 chimeras.** HeLa cells were transiently transfected with HA-tagged IMPDH chimera or wild-type constructs (as labelled) and harvested in activity assay buffer. (A) Lysates (20 µg) were processed by SDS-PAGE and analysed by western blot using anti-HA antibody. Data is representative of three independent experiments. (B) IMPDH activity was determined in cell lysates at 37°C by measuring the production of NADH as previously described [9]. Values were normalised to exogenous HA-IMPDH protein expression, as determined by western blot, and represent the mean ± SD of two independent experiments.
(TIF)

Figure S6 **Sensitivity of human IMPDH to elastase.** Representative Coomassie stained SDS-PAGE gels of (A) His-tagged IMPDH1 protein or (B) His-IMPDH2 protein incubated in *in vitro* activity assay buffer at RT with elastase (E) for the times indicated. Note: elastase is a 26 kDa protein and was not included in the zero time point. Molecular weight (MW) marker sizes are as indicated. (C) Purified His-tagged IMPDH1 or (D) IMPDH2 protein was pre-incubated with 1 mM of ligands, as indicated, and subjected to a protease protection assay. Protease protection assay reactions were resolved by SDS-PAGE and gels were either stained by coomassie or transferred onto membrane for Western blotting with the anti-panIMPDH antibody or isoform-specific antibodies (α6Core1; raised against peptide in IMPDH1 core domain and α3Sub2 raised against peptide in IMPDH2 sub domain [9]), as indicated, which were scanned in two fluorescent channels using the LI-COR Odyssey system. Panels show the individual blots and merged image (pan-IMPDH - green; isoform-specific antibodies - red).
(TIF)

Figure S7 **Dose dependent protection of IMPDH to elastase.** Representative Coomassie stained gels of a protease protection assay with His-IMPDH1 or His-IMPDH2 proteins showing the dose-dependent increase in protection from proteolysis of IMPDH2 by AMP and IMPDH1 by ATP. Concentrations are in mM.
(TIF)

Video S1 **Time-lapse of MPA induced clustering of HA-IMPDH2-GFP in live cells.** Time-lapse corresponding to Fig. 3A. After MPA addition (15∶30), Z-series (total 100) were captured at a rate of 1/80 s. The playback parameters were set at a rate of 20 frames/s condensing the 2∶13∶15 movie into 5 s.
(AVI)

Video S2 **Subset of MPA induced clustering of HA-IMPDH2-GFP in live cells.** Time-lapse corresponding to Fig. 3B and subset of Video S1.
(AVI)

Video S3 **Time-lapse of decoyinine induced clustering of HA-IMPDH2-GFP in live cells.** Time-lapse corresponding to Fig. S2A. After decoyinine addition (19∶30), Z-series (total 151) were captured at a rate of 1/90 s. The playback parameters match the real-time rate of Video S1 and Video S2, a rate of 17.9 frames/s, condensing the 4∶03∶57 movie into 8.4 s.
(AVI)

Video S4 **Time-lapse of decoyinine induced clustering of HA-IMPDH2-GFP in live cells.** Time-lapse corresponding to Fig. S2B and subset of Video S3.
(AVI)

Table S1 **Sequences of the oligonucleotides used to generate chimeras.**
(DOC)
