# Supplementary material for: Different Characteristics and Nucleotide Binding Properties of Inosine Monophosphate Dehydrogenase (IMPDH) Isoforms
Source: PLoS One. 2012 Dec 7;7(12):e51096. doi: 10.1371/journal.pone.0051096 (PMC3517587; doi:10.1371/journal.pone.0051096)
Supplement: Text S1 — Supporting information for HA-IMPDH2-GFP fusion protein. (DOCX) [file pone.0051096.s001.docx]

Text S1. **Supporting information for HA-IMPDH2-GFP fusion protein**

To investigate the dynamics of IMPDH clustering in real-time, we first generated a HA-IMPDH2-GFP chimera. We used a 16 amino-acid linker containing predominantly glycines, serines and alanines (GTGASGAGGSGGPVAT) to link IMPDH2 and GFP (Fig. S1A). Western blot analysis of cells transiently expressing HA-IMPDH2-GFP demonstrated a predominant band of the expected size (≈82 kDa) (Fig. S1B), equivalent to the sum of GFP (26 kDa) and HA-IMPDH2 (56 kDa). Enzymatic activity was not affected by addition of the GFP moiety (Fig. S1C). Immunofluorescence microscopy revealed a diffuse, cytosolic distribution in control cells (Fig. S1D). MPA induced clustering of HA-IMPDH2-GFP was apparent in cells expressing low, but not high, levels of transfected protein (Fig. S1D). Collectively these results suggested that the HA-IMPDH2-GFP construct would be suitable for characterising real-time clustering of IMPDH in live-cells expressing low levels of HA-IMPDH2-GFP. We then generated a HA-IMPDH2-GFP stable HeLa cell line (HeLa HA-IMPDH2-GFP) and selected for low expressers by FACS. This stable population of cells expressed HA-IMPDH2-GFP at around 10% of the levels of endogenous IMPDH (Fig. S1B, lane 4) and was used to visualise IMPDH clustering in live cells.
