# Supplementary material for: Different Characteristics and Nucleotide Binding Properties of Inosine Monophosphate Dehydrogenase (IMPDH) Isoforms
Source: PLoS One. 2012 Dec 7;7(12):e51096. doi: 10.1371/journal.pone.0051096 (PMC3517587; doi:10.1371/journal.pone.0051096)
Supplement: Text S2 — Supporting information mapping the elastase cleavage site of human IMPDH. (DOCX) [file pone.0051096.s002.docx]

Text S2. **Supporting information mapping the elastase cleavage site of human IMPDH.**

The elastase cleavage sites, previously described by Nimmesgern et al., (1996) [26], were confirmed to map to the catalytic flap region within the core domain of human IMPDH by Western blotting using a panel of IMPDH antibodies, including core-specific and Bateman-domain specific antibodies [9] (Fig. S6C and D). Several lines of evidence support this conclusion: i) the mobility of the major cleavage products is consistent with the predicted molecular weight of approximately 415-445 amino acids; ii) the 3Sub2 antibody, raised against an IMPDH2 peptide (residues 126-140) within the Bateman domain and specific for IMPDH2 [9], recognises the major cleavage products of His-IMPDH2 iii) the 6coreI antibody, raised against a IMPDH1 peptide (residues 418-438) within the flap region and demonstrated to preferentially detect IMPDH1 [9], recognised the upper cleavage band of His-IMPDH1 but not the lower products indicating the subsequent loss of the 6coreI antigen; iv) the cleaved products are recognised by an anti-His antibody (data not shown), indicating these species have an intact N-terminus; v) the core domain is sensitive to elastase.
