# Supplementary figures and images for: Different Characteristics and Nucleotide Binding Properties of Inosine Monophosphate Dehydrogenase (IMPDH) Isoforms
Source: PLoS One. 2012 Dec 7;7(12):e51096. doi: 10.1371/journal.pone.0051096 (PMC3517587; doi:10.1371/journal.pone.0051096)

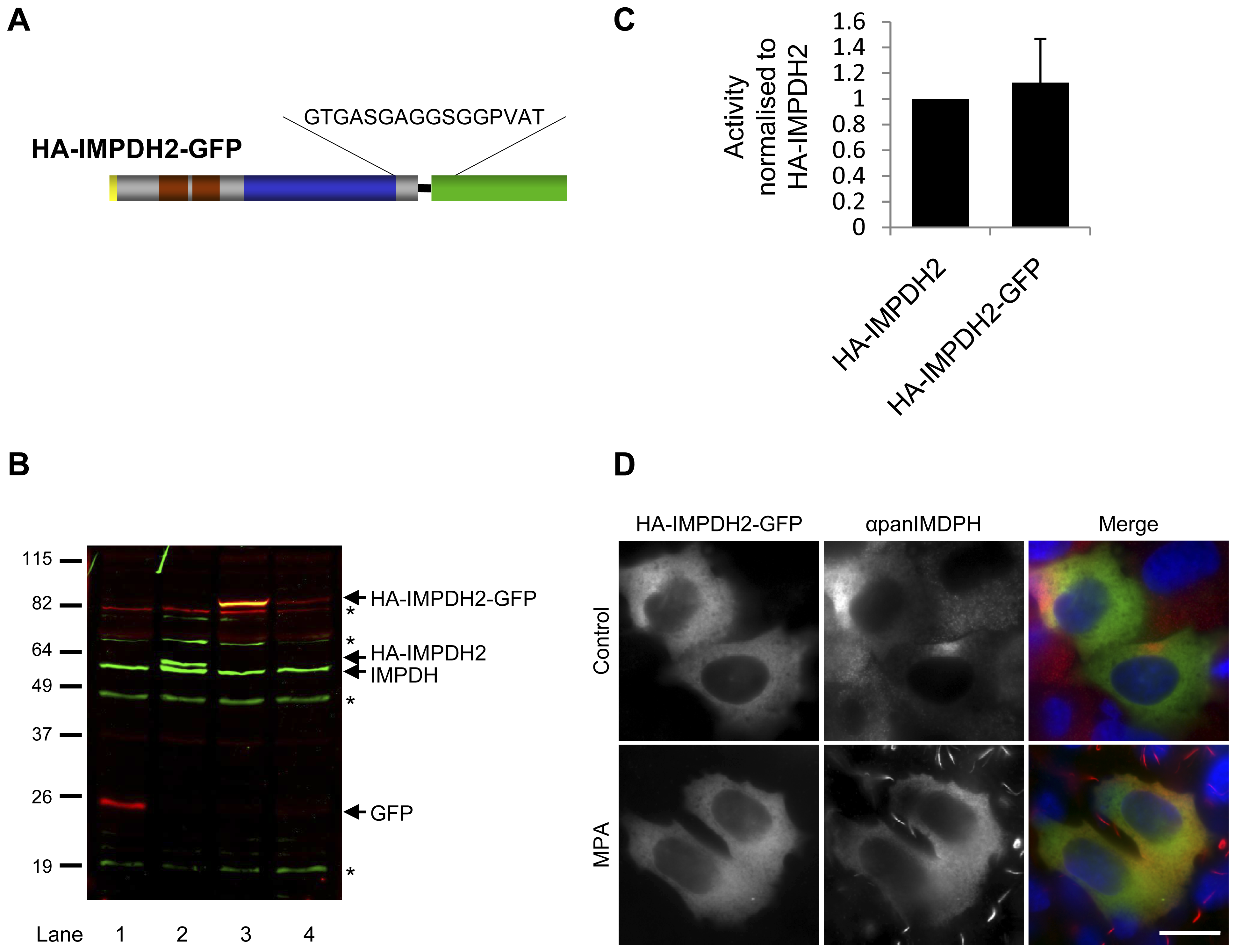

Supplement: Figure S1 — Characterisation of HA-IMPDH2-GFP fusion protein. (A) Cartoon of HA-IMPDH2-GFP fusion protein with the linker sequence indicated between IMPDH and GFP (green box). Yellow box represents the HA-tag, maroon boxes indicates the CBS domains and the catalytic domain is shown in blue. (B) Lysates (20 µg) of HeLa cells transiently expressing either GFP (lane 1), HA-IMPDH type II (lane 2) or HA-IMPDH2-GFP (lane 3) or the stable HeLa HA-IMPDH2-GFP cells (lane 4) were analysed by SDS-PAGE and western blotting. Membrane was probed with anti-GFP antibody (red) and anti-panIMPDH antibody (green). Asterisks indicate non-specific bands. (C) IMPDH activity was determined in cell lysates of HeLa cells transiently expressing HA-IMPDH2-GFP or HA-IMPDH2 or GFP alone, to control for endogenous IMPDH activity, as previously described [9]. Activity was normalised to exogenous protein expression, as determined by western blot, and the values expressed in terms of HA-IMPDH2 activity. Data represents the mean ± SD of two independent experiments. (D) Representative epifluorescence micrographs of HeLa cells transiently expressing HA-IMPDH2-GFP (green). Cells were incubated with vehicle (control) or with 2 µM MPA for 4 h. Cells were fixed, permeabilised and labelled with the anti-panIMPDH antibody and Alexa-594 secondary antibody (red) and nuclei were counterstained with DAPI (blue). Images are representative of at least three independent experiments. Scale bar = 20 µm. (TIF) [file pone.0051096.s003.tif]

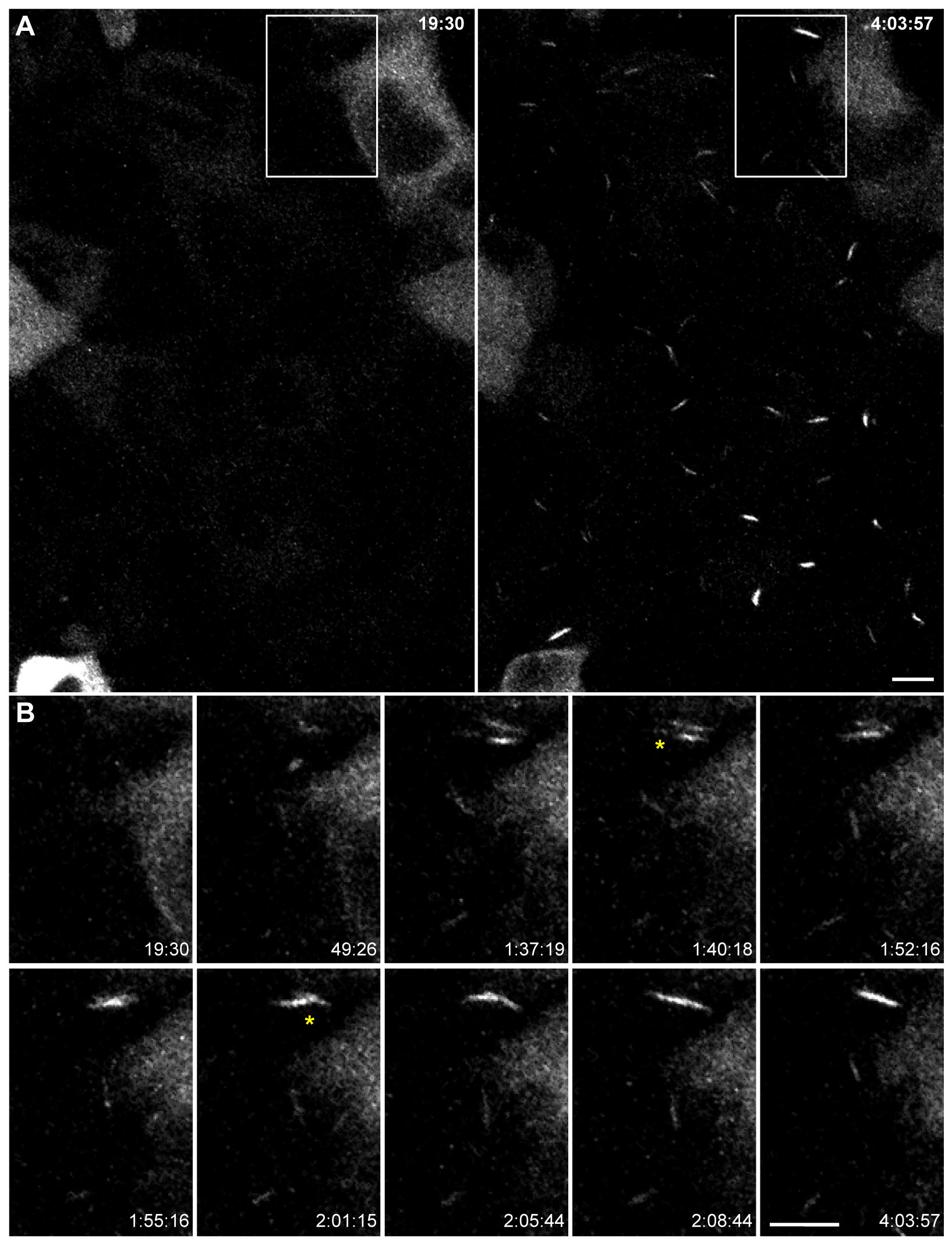

Supplement: Figure S2 — Decoyinine induced clustering of HA-IMPDH2-GFP in live cells. Images are maximum intensity projections from confocal z-series of live HeLa HA-IMPDH2-GFP cells treated with 2 mM decoyinine. Selected frames, from Video S3, are presented with the time captured relative to the addition of decoyinine recorded at the top right (h∶mm∶ss). Data is representative of three independent experiments. Scale bar = 10 µm. (A) Field of cells with low HA-IMPDH2-GFP expression, which is initially diffuse throughout the cytosol and with increasing time HA-IMPDH2-GFP clusters into spicules and macrostructures. (B) Selected frames within subset regions shown in A, corresponds to Video S4, which highlight the emergence and lateral fusion (indicated by yellow asterisk) of a HA-IMPDH2-GFP macrostructure. (TIF) [file pone.0051096.s004.tif]

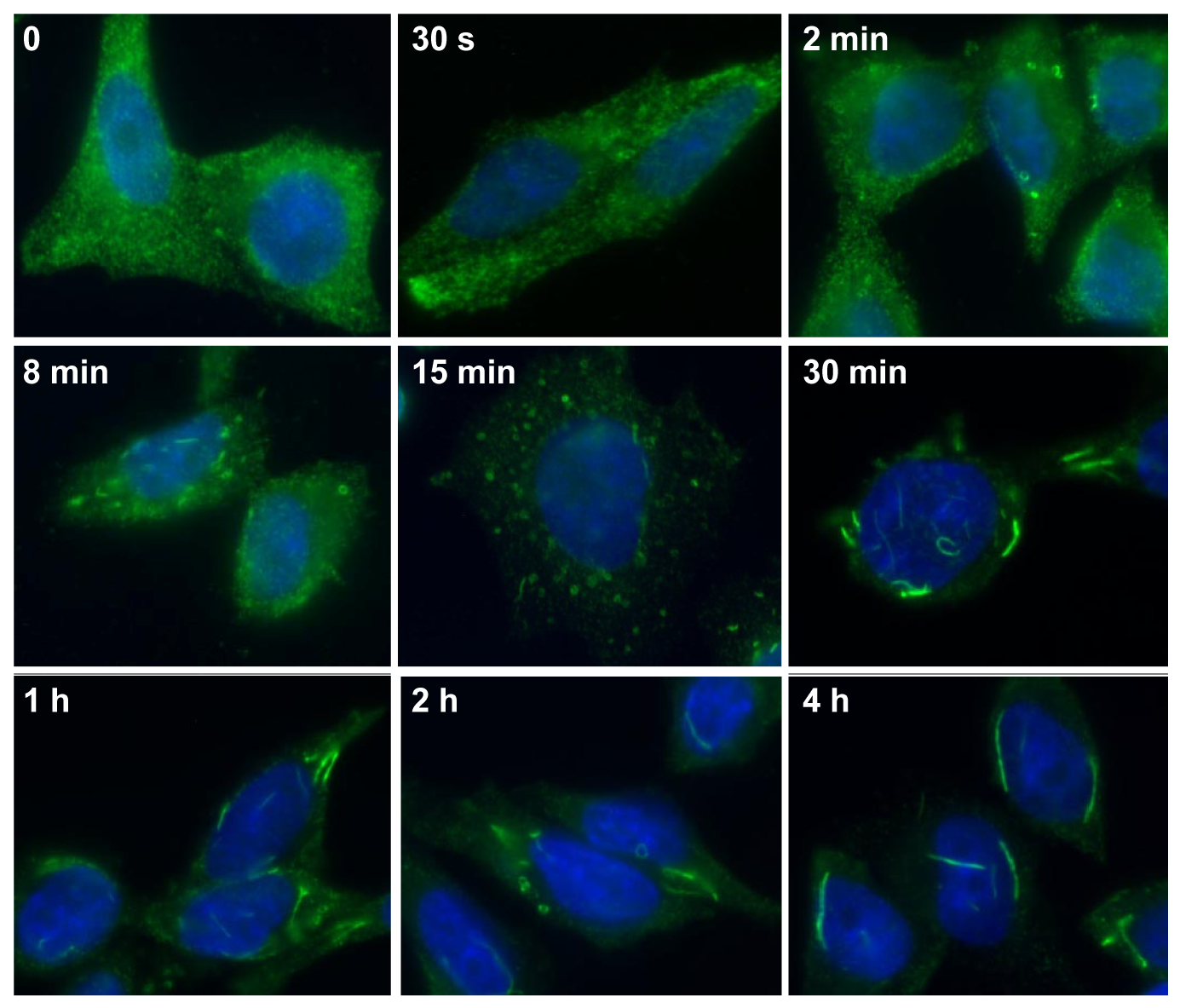

Supplement: Figure S3 — Time course of endogenous IMPDH clustering. Micrographs of CHO cells treated with vehicle (control) or 2 µM MPA for the times indicated. Cells were fixed and labelled for endogenous IMPDH (green) and nuclei were counterstained with DAPI (blue). Representative of at least three independent experiments. (TIF) [file pone.0051096.s005.tif]

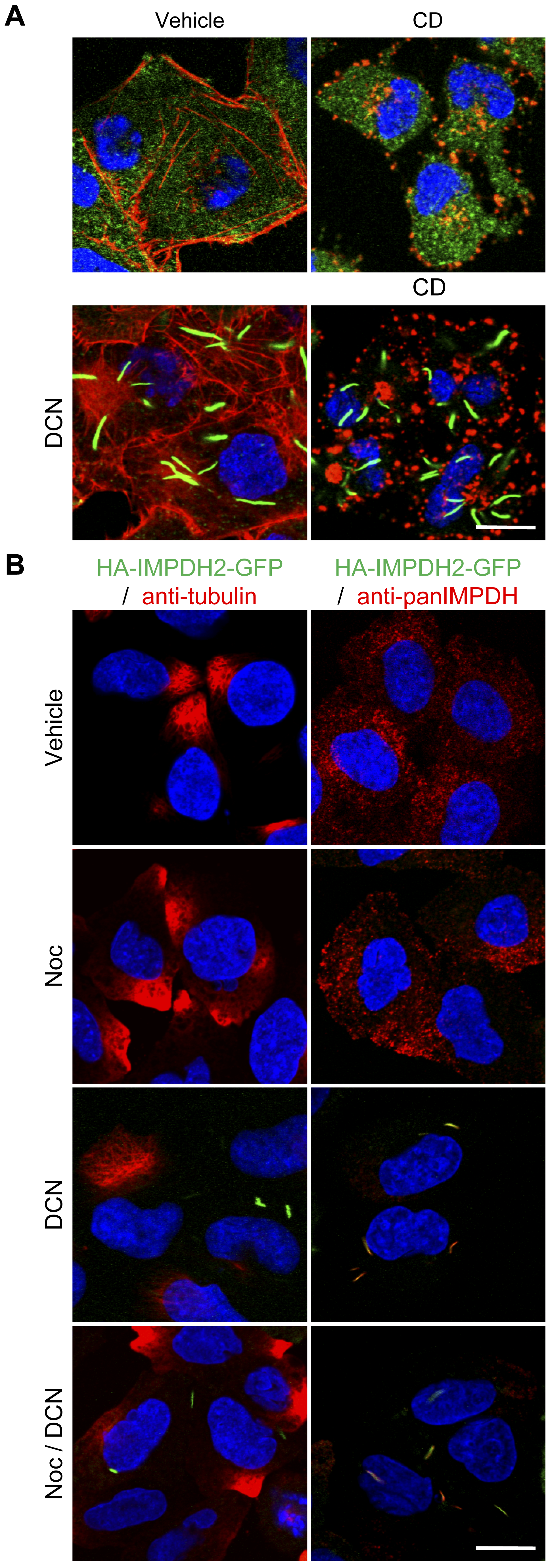

Supplement: Figure S4 — Microfilament or Microtubules alone are not required for formation of IMPDH macrostructures. Representative confocal micrographs of HeLa HA-IMPDH2-GFP cells. (A) Cells were treated with either vehicle or 4 µM cytochalasin D (CD), to discombobulate microfilaments, for 1 h 15 min prior to either fixation (top panel) or addition of 2 mM decoyinine (DCN) for an additional 3 h 30 min (bottom panel). Cells were fixed, permeabilised and labelled with the anti-panIMPDH antibody (green), filamentous actin was stained with TRITC-phalloidin (red) and nuclei were counterstained with DAPI (blue). Images are representative of at least three independent experiments. Scale bar = 20 µm. (B) Cells were treated with either vehicle or 20 µM nocodazole (Noc), to destabilise microtubules, for 1 h 15 min prior to either fixation or addition of 2 mM decoyinine (DCN) for an additional 3 h 50 min. Cells were fixed, permeablised and labelled with the anti-tubulin antibody (left hand panel; red) or anti-panIMPDH antibody (right hand panel; red) and nuclei were counterstained with DAPI (blue). In green is the low fluorescence of the HA-IMPDH2-GFP. Images are representative of at least two independent experiments. Scale bar = 20 µm. (TIF) [file pone.0051096.s006.tif]

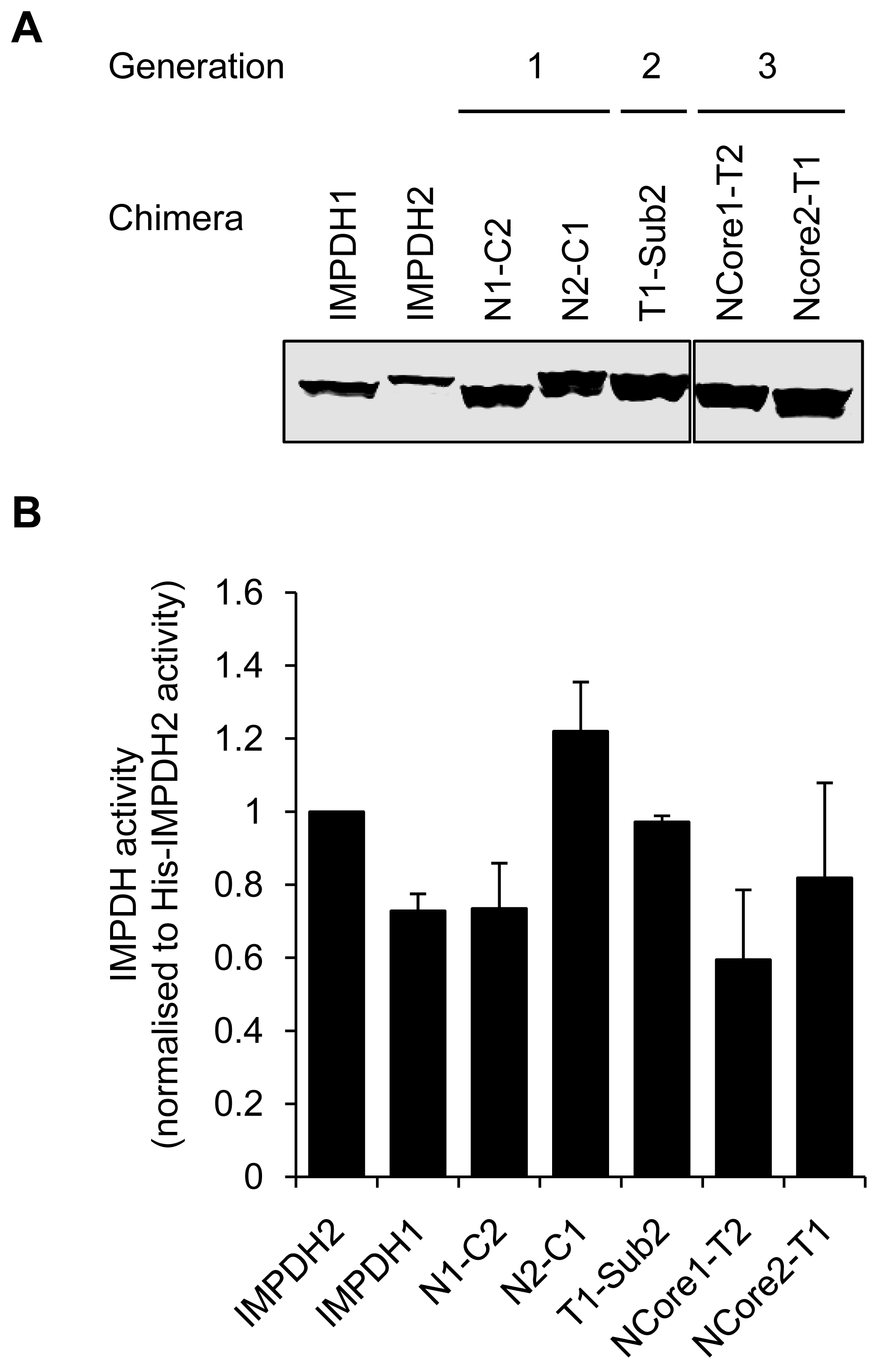

Supplement: Figure S5 — Expression and activity of IMPDH1/IMPDH2 chimeras. HeLa cells were transiently transfected with HA-tagged IMPDH chimera or wild-type constructs (as labelled) and harvested in activity assay buffer. (A) Lysates (20 µg) were processed by SDS-PAGE and analysed by western blot using anti-HA antibody. Data is representative of three independent experiments. (B) IMPDH activity was determined in cell lysates at 37°C by measuring the production of NADH as previously described [9]. Values were normalised to exogenous HA-IMPDH protein expression, as determined by western blot, and represent the mean ± SD of two independent experiments. (TIF) [file pone.0051096.s007.tif]

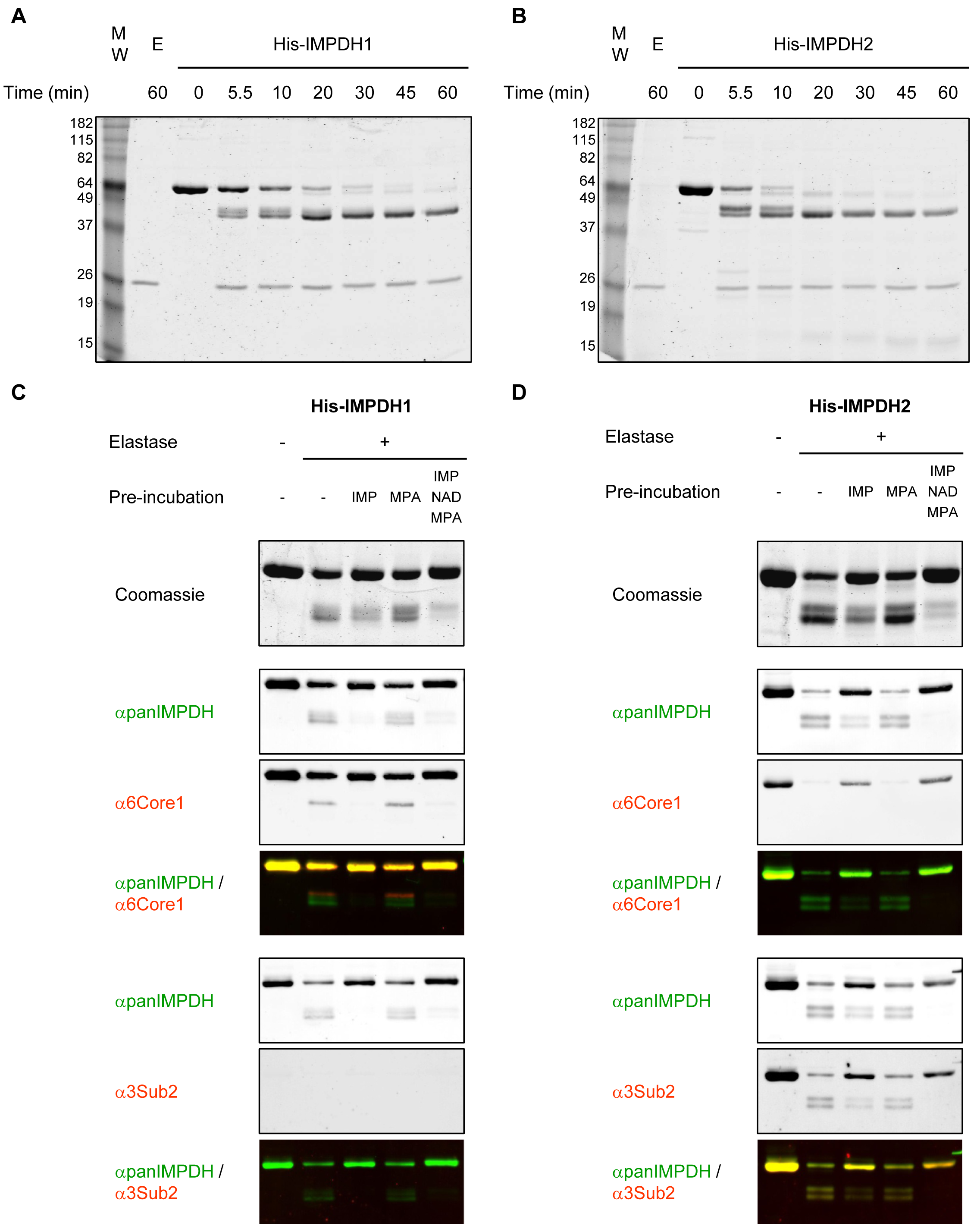

Supplement: Figure S6 — Sensitivity of human IMPDH to elastase. Representative Coomassie stained SDS-PAGE gels of (A) His-tagged IMPDH1 protein or (B) His-IMPDH2 protein incubated in in vitro activity assay buffer at RT with elastase (E) for the times indicated. Note: elastase is a 26 kDa protein and was not included in the zero time point. Molecular weight (MW) marker sizes are as indicated. (C) Purified His-tagged IMPDH1 or (D) IMPDH2 protein was pre-incubated with 1 mM of ligands, as indicated, and subjected to a protease protection assay. Protease protection assay reactions were resolved by SDS-PAGE and gels were either stained by coomassie or transferred onto membrane for Western blotting with the anti-panIMPDH antibody or isoform-specific antibodies (α6Core1; raised against peptide in IMPDH1 core domain and α3Sub2 raised against peptide in IMPDH2 sub domain [9]), as indicated, which were scanned in two fluorescent channels using the LI-COR Odyssey system. Panels show the individual blots and merged image (pan-IMPDH - green; isoform-specific antibodies - red). (TIF) [file pone.0051096.s008.tif]

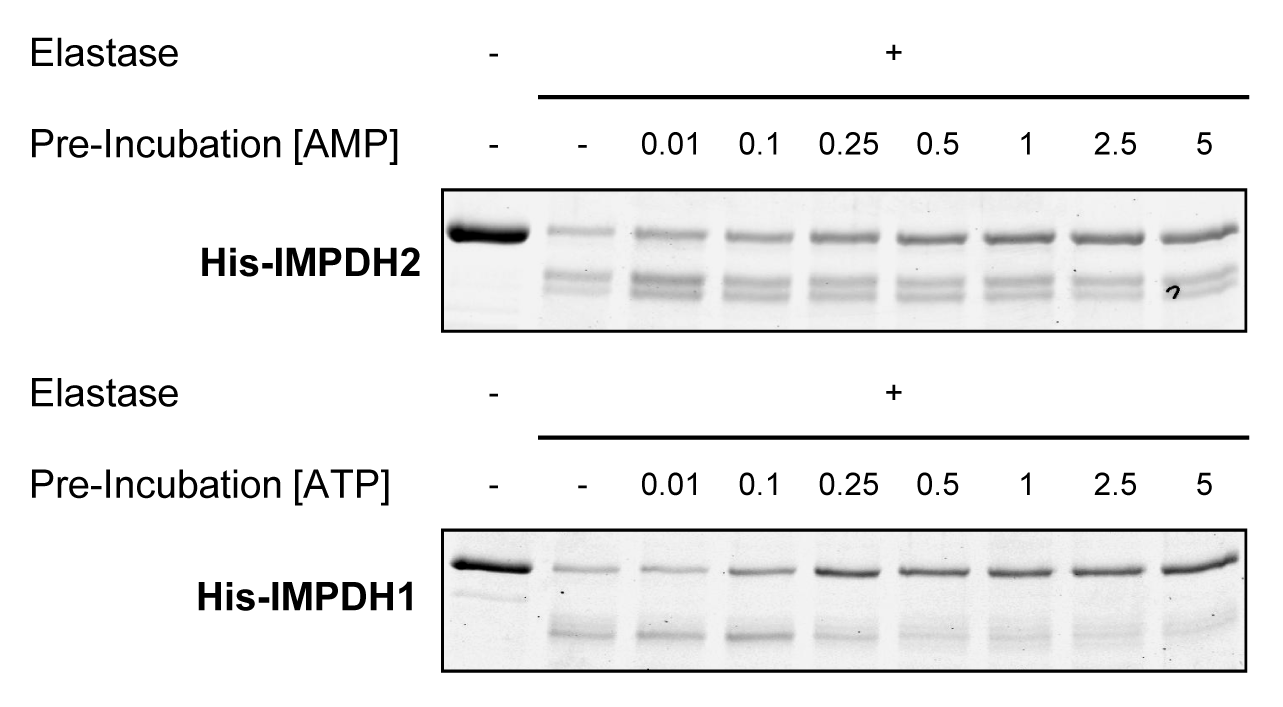

Supplement: Figure S7 — Dose dependent protection of IMPDH to elastase. Representative Coomassie stained gels of a protease protection assay with His-IMPDH1 or His-IMPDH2 proteins showing the dose-dependent increase in protection from proteolysis of IMPDH2 by AMP and IMPDH1 by ATP. Concentrations are in mM. (TIF) [file pone.0051096.s009.tif]
