# Supplementary material for: Different Characteristics and Nucleotide Binding Properties of Inosine Monophosphate Dehydrogenase (IMPDH) Isoforms
Source: PLoS One. 2012 Dec 7;7(12):e51096. doi: 10.1371/journal.pone.0051096 (PMC3517587; doi:10.1371/journal.pone.0051096)
Supplement: Table S1 — Sequences of the oligonucleotides used to generate chimeras. (DOC) [file pone.0051096.s014.doc]

**Table S1.** Sequences of the oligonucleotides used to generate chimeras

| **Final Construct** | **PCR** | **Template** | **F/R** | **Primer 5’-3’** |
| --- | --- | --- | --- | --- |
| pcDNA5/FRT/TO HA-Core2 | A | pcDNA5/FRT/TO HA-IMPDH2 | F | GGTGTAAGCTTGCCACCATGTACCCATACGATGTGCCAGATTACGCTGCCGACTACCTGATTAGTGG |
|  |  |  | R | CCCACACAGACCAGTACGCATCTTCACTTTCCGAAC |
|  | B | pcDNA5/FRT/TO HA-IMPDH2 | F | AAAGTGAAGATGCGTACTGGTCTG TGT GGGGCAGCC |
|  |  |  | R | GGTGGTTCTAGATCAGAAAAGCCGCTTCTC |
| pcDNA5/FRT/TO HA-Sub2 |  | pcDNA5/FRT/TO HA-IMPDH2 | F | GCCACCATGTACCCATACGATGTGCCAGATTACGCTTTCCAGGCCAATGAAGTTCGG |
|  |  |  | R | CAGCTGTTTCTTGGCATC |
| pcDNA5/FRT/TO HA-N1-C2 | A | pcDNA5/FRT/TO HA-IMPDH1 | F | GGTGGTAAGCTTGCCACCATGTACCCATACGATGTGCCAGATTACGCTGCGGACTACCTGATCAGCG |
|  |  |  | R | cccacacagcagctgcttctggga |
|  | B | pcDNA5/FRT/TO HA-IMPDH2 | F | gaagcagctgCTGTGTGGGGCAGCC |
|  |  |  | R | GGTGGTTCTAGATCAGAAAAGCCGCTTCTC |
| pcDNA5/FRT/TO HA-N2-C1 | A | pcDNA5/FRT/TO HA-IMPDH2 | F | GGTGGTAAGCTTGCCACCATGTACCCATACGATGTGCCAGATTACGCTGCCGACTACCTGATTAGTGG |
|  |  |  | R | cccacagagcagctgtttcttggc |
|  | B | pcDNA5/FRT/TO HA-IMPDH1 | F | gaaacagctgctctgtggggcagctgtg |
|  |  |  | R | GGTGGTTCTAGATCAGTACAGCCGCTTTTCG |
| pcDNA5/FRT/TO HA-T1-Sub2 | A | pcDNA5/FRT/TO HA-IMPDH1 | F | GGTGGTAAGCTTGCCACCATGTACCCATACGATGTGCCAGATTACGCTGCGGACTACCTGATCAGCG |
|  |  |  | R | gttcatatttcttgaccttccgcaccTCG |
|  | B | pcDNA5/FRT/TO HA-N2-C1 | F | cggaaggtcaagaaatatgaacagggattc |
|  |  |  | R | GGTGGTTCTAGATCAGTACAGCCGCTTTTCG |
| pcDNA5/FRT/TO HA-NCore1-T2 | A | pcDNA5/FRT/TO HA-IMPDH1 | F | GGTGGTAAGCTTGCCACCATGTACCCATACGATGTGCCAGATTACGCTGCGGACTACCTGATCAGCG |
|  |  |  | R | gttcatatttcttgaccttccgcaccTCG |
|  | B | pcDNA5/FRT/TO HA-IMPDH2 | F | cggaaggtcaagaaatatgaacagggattc |
|  |  |  | R | GGTGGTTCTAGATCAGAAAAGCCGCTTCTC |
| pcDNA5/FRT/TO HA-NCore2-T1 | A | pcDNA5/FRT/TO HA-IMPDH2 | F | GGTGGTAAGCTTGCCACCATGTACCCATACGATGTGCCAGATTACGCTGCCGACTACCTGATTAGTGG |
|  |  |  | R | CCCTGTTCAAACTTCTTCACTTTCCGAACTTC |
|  | B | pcDNA5/FRT/TO HA-IMPDH1 | F | CGGAAAGTGAAGAAGTTTGAACAGGGC |
|  |  |  | R | GGTGGTTCTAGATCAGTACAGCCGCTTTTCG |
